# Trace Metal Acquisition by Marine Heterotrophic Bacterioplankton with Contrasting Trophic Strategies

**DOI:** 10.1128/AEM.03128-15

**Published:** 2016-02-19

**Authors:** Shane L. Hogle, J. Cameron Thrash, Chris L. Dupont, Katherine A. Barbeau

**Affiliations:** a Geosciences Research Division, Scripps Institution of Oceanography, La Jolla, California, USA; b Department of Biological Sciences, Louisiana State University, Baton Rouge, Louisiana, USA; c J. Craig Venter Institute, La Jolla, California, USA; Goethe University Frankfurt am Main

## Abstract

Heterotrophic bacteria in the SAR11 and Roseobacter lineages shape the marine carbon, nitrogen, phosphorous, and sulfur cycles, yet they do so having adopted divergent ecological strategies. Currently, it is unknown whether these globally significant groups partition into specific niches with respect to micronutrients (e.g., trace metals) and how that may affect marine trace metal cycling. Here, we used comparative genomics to identify diverse iron, cobalt, nickel, copper, and zinc uptake capabilities in SAR11 and Roseobacter genomes and uncover surprising unevenness within and between lineages. The strongest predictors for the extent of the metal uptake gene content are the total number of transporters per genome, genome size, total metal transporters, and GC content, but numerous exceptions exist in both groups. Taken together, our results suggest that SAR11 have strongly minimized their trace metal uptake versatility, with high-affinity zinc uptake being a unique exception. The larger Roseobacter genomes have greater trace metal uptake versatility on average, but they also appear to have greater plasticity, resulting in phylogenetically similar genomes having largely different capabilities. Ultimately, phylogeny is predictive of the diversity and extent of 20 to 33% of all metal uptake systems, suggesting that specialization in metal utilization mostly occurred independently from overall lineage diversification in both SAR11 and Roseobacter. We interpret these results as reflecting relatively recent trace metal niche partitioning in both lineages, suggesting that concentrations and chemical forms of metals in the marine environment are important factors shaping the gene content of marine heterotrophic Alphaproteobacteria of the SAR11 and Roseobacter lineages.

## INTRODUCTION

The bioactive trace metals manganese, iron, cobalt, nickel, copper, and zinc are important enzyme cofactors for microbially mediated processes that drive nutrient cycling in the ocean. Marine phytoplankton and heterotrophic bacterioplankton require these metals for important cellular metabolisms (1), with some closely related species having very different cellular metal requirements and metal-induced physiological responses (2). At larger scales, the spatial and temporal distributions of certain trace metals can have profound ecosystem-wide consequences (3). The concentrations of the bioactive trace elements are generally very low in the open ocean due to the isolation of the pelagic ocean from terrestrial inputs and in some cases limited solubility (e.g., Fe). The chemical speciation of Fe, Cu, and potentially Co in seawater is highly dependent upon each metal's propensity to interact with heterogeneous organic ligands (4–6). Ni and Zn also interact with ligands in seawater (7, 8), and natural organic ligands in seawater appear to minimally interact with Mn (1). The dilute concentrations of marine trace metal species, their variable redox states, and vast structural diversity likely provide a spectrum of trace metal niches for marine heterotrophic bacteria and phytoplankton.

There are many currently identified trace metal uptake systems (9, 10), and here we briefly introduce the major known pathways for metal uptake in Gram-negative bacteria (see Table S1 in the supplemental material). It should be noted that these pathways have primarily been characterized in copiotrophic, nonmarine organisms, many of which are host pathogens. In the context of the marine environment it is probable that transport systems with little or no functional precedent are employed by marine bacteria, especially those from unique, taxonomically underrepresented, and unculturable lineages.

Inorganic Fe^3+^ is transported through the bacterial inner membrane by ATP binding cassette transporters (ABCT). ABCTs are transmembrane, ATP-dependent transport proteins comprised of a periplasmic substrate-binding protein, a permease, and an ATP-binding component. Inorganic Fe^2+^ is transported by four inner-membrane transporter families. NRAMP-like proteins facilitate Fe^2+^ and Mn^2+^ transport in some bacteria (11), while the ZIP family can import Fe^2+^, Mn^2+^, Zn^2+^, and Co^2+^ (12). FTR1-like proteins can function as Fe^2+^ transporters (13), while the FeoAB system is an Fe-specific bacterial permease (14). Many Fe transporters are regulated by the ferric uptake repressor protein (Fur) (15), a transcription factor that utilizes Fe^2+^ as a corepressor. The Fur protein represses transcription by first binding Fe^2+^ and then binding to a conserved 19-bp inverted repeat called a Fur box. Iron regulatory motifs, called iron-rhodo boxes, with a palindromic repeat different from but related to that of the Fur box have previously been predicted to be upstream of most iron transporters in 12 different Roseobacter genomes (16).

Siderophores and heme/hemoproteins are two major organic Fe forms utilized by bacteria. Hydroxamate and catecholate functional groups are two structural motifs found in siderophores, and both chemical classes are biosynthesized in nonribosomal peptide synthetase (17) (NRPS) or NRPS-independent pathways (18). TonB-dependent transporters (TBDTs) import Fe-bound siderophores across the bacterial outer membrane, while ABCTs move siderophores through the inner membrane. Periplasmic substrate-binding proteins of the *fatB* family transport catecholate siderophores such as enterobactin and anguibactin (19), while *fhuD* substrate binding proteins are specific for hydroxamate siderophores (20). In the cytoplasm, siderophore-bound Fe is reduced by siderophore-interacting proteins (SIPs) which release Fe^2+^ to be utilized in downstream cellular processes (21). In Gram-negative bacteria, heme is imported by heme-specific TBDTs coupled with heme-specific ABCT systems in a manner analogous to that of siderophores. Heme uptake ABCTs utilize a characteristic substrate-binding protein, HutB. A cytoplasmic binding protein, HmuS, is also typically encoded within characterized heme uptake operons, although its exact function is currently unresolved (22).

In model organisms Cu, Zn, Co, and Ni move through porins and TBDTs at the outer membrane and through ABCTs and other transmembrane proteins at the inner membrane. Some TroA-family ABCT substrate binding proteins (23) participate in the uptake of Mn^2+^ (24), Zn^2+^ (25), and vitamin B_12_ (26). Ni complex uptake has been demonstrated to occur through a non-TroA ABCT system, NikA (27). Other Ni and Co permeases include the secondary transporter family NiCoT (Ni and Co) (28), the related HupE/UreJ (29) family (Ni), and the CbiMNQO/NikMNQO systems, which are hypothesized to transport Co and Ni, respectively (30). CbtA is predicted to be an inner-membrane ion channel specific for Co (31), although this is yet to be experimentally confirmed. CorA inner-membrane ion channels were initially characterized as Mg^2+^ transporters, but recent work demonstrated that the family can be highly selective for Co^2+^ (32). As mentioned before, NRAMP and ZIP transporters can operate as generalized divalent metal transporters. Cu tolerance in certain bacteria is known to be facilitated by P_1B_-type ATPases, which include both efflux and import transporters. The Cu P_1B_-type ATPase system *copA* (33) has been identified as a Cu efflux transporter essential for Cu resistance. The same protein family is also required for the biosynthesis of multiple Cu-containing enzymes in Rubrivivax gelatinosus, implicating it in Cu import (34). Some bacteria potentially use metallochaperones of the *copZ* family (35) to manage intracellular Cu levels (36).

Heterotrophic marine bacteria are commonly divided into two ecological categories: those that are streamlined oligotrophs (37) and those that are copiotrophs (38, 39), although a continuum certainly exists between these two extremes. Genome-streamlined bacteria appear to be “background adapted” and succeed by utilizing the persistent but extremely low background concentrations of nutrients under relatively static conditions, whereas metabolically variable copiotrophs are “patch adapted” and exploit transient nutrient hotspots and variable microscale habitats. Although these coarse divisions do not capture the full complexity of microbial niche space and evolution, they have been shown to be useful in conceptualizing marine microbial ecosystems (39, 40). Patch-adapted marine bacteria rapidly colonize particles and other surfaces and are thought to be primarily responsible for hydrolyzing and degrading structurally complex organic matter while liberating smaller and more labile molecules (41, 42). In contrast, background-adapted organisms are primarily free living, do not readily associate with surfaces, and have generally low extracellular enzymatic activity. Both patch- and background-adapted organisms use membrane-bound transporters to extract specific molecules from their immediate environment in order to acquire nutrients. These bacterial uptake and degradation processes may alter the microscale trace metal reactivity landscape by liberating metals from sinking particles, modifying metal speciation in the dissolved phase, or selectively removing certain metal complexes.

The Roseobacter and SAR11 lineages are two diverse and highly abundant groups of marine Alphaproteobacteria that generally represent patch-adapted and background-adapted ecological strategies, respectively (43). Although roseobacters do not neatly cluster into ecotypes, many cultured representatives have extensive and diverse gene inventories for carbon and energy acquisition consistent with a patch-adapted lifestyle. Recent evidence suggests that some uncultivated roseobacters have lifestyles more consistent with background-adapted organisms (44, 45). Other roseobacters frequently dominate the bacterial community on particles (46) and have been shown to be highly enzymatically active (47). In contrast, the SAR11 lineage is comprised of different ecotypes (48), its members have small streamlined genomes with low GC content and comparatively limited metabolic capability (49), and they do not associate with particles or surfaces. Combined, SAR11 and Roseobacter can comprise up to 40% of total bacteria in marine surface waters (50).

Gene content and diversity have been shown to reflect microbial adaptation at ocean basin scales (51–53) as well as at microscales (54, 55), but these adaptations have largely been explored only for nutrients like nitrogen and phosphorous. Here, we analyzed genomes from two extremes of the patch-adapted–background-adapted continuum (exemplified by Roseobacter and SAR11 genomes, respectively) in order to explore how generalized ecological strategy shapes the specific genomic capabilities for trace metal uptake. First, we surveyed the extent and diversity of known Mn, Fe, Co, Ni, Cu, and Zn uptake systems in 42 Roseobacter genomes and 22 SAR11 genomes. Second, we evaluated gradations of uptake capabilities among genomes and examined lineage evolutionary history as a structuring factor. Finally, we explored relationships between the genetic potential for trace metal uptake, environmental factors, and genomic features and how these relationships are organized in a patch-adapted-versus-background-adapted framework. Roseobacter and SAR11 are not the only bacterial lineages representing this ecological paradigm (e.g., the background-adapted SAR86 lineage [56] and patch-adapted Alteromonadales [57]), and caution should be taken when the trends reported in this work are extrapolated to other marine bacterial groups. However, this study contextualizes marine bacterial trace metal transporters within evolutionary and ecological frameworks. Although the phylogenetic representation here is limited to two dominant marine groups from the Alphaproteobacteria, we expect that our conclusions may have broader implications for other marine heterotrophic bacterial groups.

## MATERIALS AND METHODS

In addition to the supplemental material files associated with this article, complete supplemental text, code, and data sets are available at http://dx.doi.org/10.6084/m9.figshare.1533034.

### Genomic sequence data and genome classification schemes.

All microbial genomes and associated metadata were obtained from the IMG database (58) (April 2015). Roseobacter genomes were selected from IMG to reflect the content of Roseobase (www.roseobase.org), which is a comprehensive genomic resource for marine Roseobacter strains. All publically available SAR11 genomes available in IMG with a genome completeness greater than 90% were included in this study. Genome completeness and integrity of all isolates were assessed using the CheckM pipeline (59). Lifestyle (surface associated or free living), isolation location (Atlantic Ocean, Pacific Ocean, Indian Ocean, or polar seas), and isolation land proximity (coastal or pelagic) were assigned when the data were available in IMG or the primary literature. For details, refer to the text and data sets in the supplemental material.

### Functional prediction and annotation.

The metal transport systems used for searches in Roseobacter and SAR11 genomes (see Table S1 and Data Set S2 in the supplemental material) include the majority of currently characterized/predicted metal transport systems (9, 10). Metal transporters were identified using the NCBI conserved domain database (60). Orthologs were identified by RPS-BLAST hits (e value < 10^−5^) to conserved domain database models, and only bidirectional reciprocal “specific hits” to domain models (those above significance threshold values) were retained. Nineteen DNA base pair segments of predicted Roseobacter Fur boxes were obtained from a prior study (16) and used to search for new Fur boxes in the remaining 42 Roseobacter genomes using BLASTN. Fur boxes were identified in SAR11 genomes by homology to a 15-base inverted-repeat motif (61). TBDTs were clustered by sequence similarity using the Markov clustering algorithm (MCL) (62). Fisher's exact test was used to test if protein families were enriched in TBDT gene neighborhoods (10 genes upstream and downstream of TBDT) as described earlier (63). For details, see the text and data sets in the supplemental material.

### Phylogenetic tree inference and phylogenetic conservation of functional traits.

Of 28 composition-homogenous orthologous protein families (64), 26 (excluding COG0238 and COG0522) were identified in the 64 genomes (see Data Set S4 in the supplemental material). Amino acid sequences in each orthologous set were aligned using MUSCLE (65), culled using Gblocks (66), and concatenated. Phylogenetic inference was performed with RAxML HPC v7.7.6 (67). Phylogenetic clustering of metal uptake categories was investigated using Fritz and Purvis' phylogenetic dispersion metric (*D*) (68) and trait depth (τ_*D*_) from the consenTRAIT algorithm (69). For details, see the text and data sets in the supplemental material.

### Multivariate statistics and correlation analysis.

Multivariate statistics were performed using the VEGAN (70) package in R. Patterns of metal uptake genes among genomes were explored using both principal coordinates analysis (PCA) and nonmetric multidimensional scaling (NMDS) using the Bray-Curtis dissimilarity index. Classification schemes and genome features were fitted as vectors to the ordination using the envit VEGAN function with 999 permutations to assess significance. Spearman's rank correlation coefficient was used to test for correlations between transporter abundances, and Pearson's chi-squared test was used for categorical data. All multiple tests were corrected using the Benjamini-Hochberg method for controlling the false discovery rate. For details, see the text and data sets in the supplemental material.

## RESULTS

### Uptake systems for inorganic Fe.

**FIG 1:**
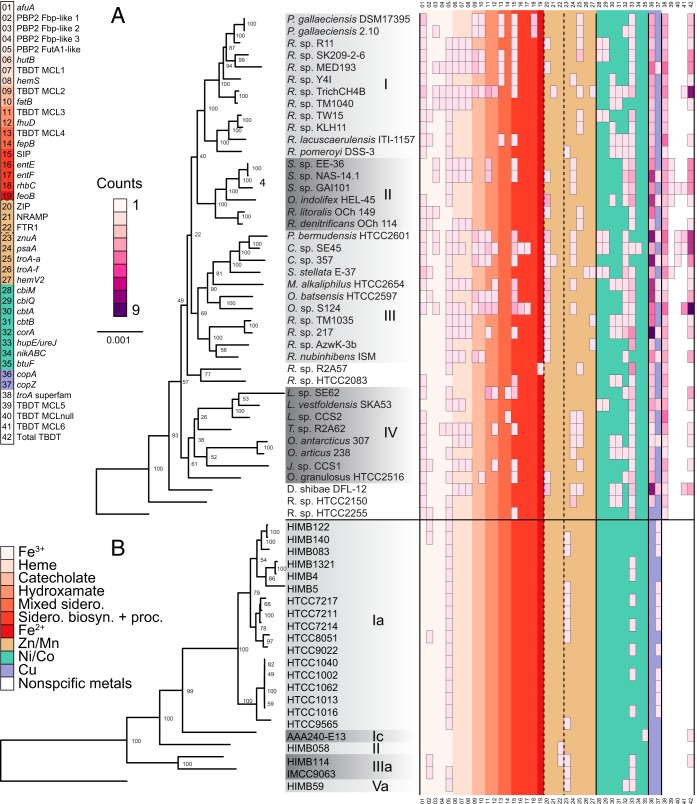
Maximum-likelihood phylogenies of 42 Roseobacter genomes (A) and 22 SAR11 genomes (B). Node values indicate bootstrap values from 1,000 resamplings. The scale bar represents 0.001 substitution per sequence position in both trees. Shaded boxes beneath Roseobacter names in panel A indicate the four major clades as presented in reference 43, while those in panel B denote five major clades in the SAR11 group. The central grid represents the absolute abundance of each respective metal uptake system per genome, the values of which are represented by color as shown in the scale labeled “Counts.” Classes of metal uptake systems are partitioned by color, as shown in the lower left corner. Specific metal uptake genes are referenced by number and color on the upper left, where numbers correspond to rows in the central grid. NRAMP, FTR1, and ZIP transporters interact with Fe, Zn, and Mn and are indicated with dashed lines.

We used the solute-binding protein, the most divergent and informative component of the system (71), to discriminate the particular metal substrate of ABCTs. In the case of Fe^3+^, there are genes for five different families of ferric solute-binding proteins across the genomes (Fig. 1; also, see Table S1 in the supplemental material). All Roseobacter genomes contain genes for Fe^3+^ ABCTs, and 70% of the genomes have genes for at least two different solute binding families. All SAR11 genomes contain Fe^3+^ ABCT genes, with the exception of the genomes of strains AAA240-E13 (clade Ic, isolated from 770 m at Station ALOHA) and HIMB058 (clade II, isolated from Kāne'ohe Bay, HI) (Fig. 1). Approximately 80% of SAR11 genomes contain single copies of genes for ferric solute-binding protein families, with three genomes in clade Ia and one in IIIa having two copies. The most common type of ABCT solute-binding protein encoded by all genomes is the FutA1-like protein. Dedicated systems for Fe^2+^ transport are rare in the Roseobacter clade. Of the genomes surveyed, 37% contain proteins in the ZIP family, but ZIP proteins transport metals other than Fe. The Fe-specific *feoAB* system is found only in the genome of Roseobacter sp. strain R2A57, NRAMP gene homologs are present in the genomes of *Citreicella* sp. strain 357 and Ruegeria sp. strain TrichCH4B, and no Roseobacter genome contains orthologs of the FTR1 gene (Fig. 1). Genes for Fe^2+^ uptake systems are also rare in SAR11 genomes. No SAR11 genome contains genes for ZIP, FeoAB, or NRAMP uptake systems, but the deeply branching genomes of HIMB058 and HIMB114 both have FTR1 permease genes (Fig. 1).

### Fur proteins and binding sites.

We predict approximately 90 new iron-rhodo box transcription factor binding sites (sequences and associated loci are available in Data Set S3 in the supplemental material) in Roseobacter genomes using the previously identified iron-rhodo box palindromic motif (16). In this expanded work, many but not all Roseobacter iron uptake genes are preceded by the iron-rhodo box motif. Seventy percent of Roseobacter genomes appear to have at least one Fe^3+^ ABCT under Fur regulation, and many Roseobacter TBDTs are preceded by iron-rhodo boxes (see Table S2 in the supplemental material). In the case of SAR11, Fur box motifs generally match a 15-bp (7-1-7) Fur box inverted repeat (61). Only the HTCC7217, HTCC7211, HIMB59, and AA240-E13 genomes are missing apparent Fur box motifs, and Fur box motifs in the remaining genomes always precede components of the Fe^3+^ ABCT uptake system as well as FTR1 permeases. Regardless, it appears that all Roseobacter and SAR11 genomes have at least one gene for a Fur-like protein, with most Roseobacter genomes containing multiple copies.

### Siderophore and heme uptake.

We classified 34 Roseobacter TBDTs as siderophore transporters based on each TBDT’s colocalization with *fatB*, *fhuD*, genes encoding siderophore-interacting proteins, and putative siderophore biosynthesis genes (see Table S2 and Data Set S5 in the supplemental material). We also identified 18 Roseobacter genomic regions as potential heme uptake systems based on the colocalization of *hmuS*, *hutB*, and TBDT genes. Many Roseobacter genomes contain multiple different siderophore uptake genetic loci, whereas none contain multiple copies of genes for heme uptake systems. No SAR11 genomes surveyed here contain systems for siderophore or heme uptake. It appears that in total, 45% of Roseobacter genomes contain the potential for exogenous heme utilization, while 40% contain the potential for siderophore uptake.

The 34 putative siderophore TBDTs cluster by sequence similarity into three different groupings (MCL2, MCL3, and MCL4) that are largely consistent with the family of ABCT substrate binding proteins present nearby. Significant enrichment of *fatB* within ±10 genes of MCL2 suggests that MCL2 is involved in the uptake of catecholate-like siderophores, while enrichment of *fhuD* and siderophore-interacting proteins within ±10 genes of MCL3 suggests that this cluster participates in hydroxamate-like siderophore uptake (see Table S2 in the supplemental material). MCL4 gene neighborhoods are enriched in siderophore-interacting proteins, substrate-binding proteins assignable only at the superfamily level, and occasionally either FatB or FhuD, suggesting that it is involved with the uptake of siderophores of an unclear structural class. Iron-rhodo box transcription binding sites are upstream of many but not all of these siderophore TBDTs, and the catecholate and mixed-siderophore clusters (MCL4 and MCLnull) have the lowest percentage of TBDTs with these sites (see Table S2 in the supplemental material). In addition to the 34 siderophore TBDTs identified by gene neighborhood alone, eight other TBDTs in ambiguous neighborhoods also cluster with MCL2, MCL3, and MCL4. These eight TBDTs all have upstream iron-rhodo boxes, suggesting that they may have a role in siderophore transport or the transport of unknown Fe complexes.

### Siderophore biosynthesis.

Putative siderophore biosynthesis clusters are present in four Roseobacter genomes and no SAR11 genomes (Fig. 1; also, see Table S1 in the supplemental material). Both the P. gallaeciensis 2.10 and P. gallaeciensis 17395 genomes contain clusters with NRPS-independent type siderophore biosynthesis genes, which are exclusive to the biosynthesis of siderophore secondary metabolites (18). In addition, the genomes of *Citreicella* sp. strain SE45 and *Oceanicola* sp. strain S124 have TBDTs colocalized with genes containing domain-specific hits to the genes for enterobactin synthase subunits E and F (NRPS) and other genes involved in siderophore uptake, biosynthesis, and regulation. Tripartite ATP-independent periplasmic (TRAP) transporters are present within two putative siderophore biosynthesis and uptake clusters, suggesting a potentially unrecognized role for TRAP transporters in siderophore uptake (see Fig. S1 and the text in the supplemental material).

### Uptake systems for Mn, Zn, Co, Cu, and Ni.

The number of experimentally characterized TBDT systems for trace metals other than Fe is small, and we observed no genes for TBDTs unambiguously related to Ni, Co, or Cu transport in Roseobacter and SAR11 genomes. We therefore focused our searches on better-known inner-membrane Ni, Co, Cu, Mn, and Zn transporters (Fig. 1; also, see Table S1 in the supplemental material). No SAR11 genomes but all 42 Roseobacter genomes contain genes for at least one helical backbone solute-binding protein assignable only at the *troA* superfamily level. The *troA* subfamilies present in Roseobacter genomes are *psaA* (38%), *troA*-a (38%), *hemV2* (10%), *troA*-f (2%), and *znuA* (2%). Eighty-three percent of Roseobacter genomes have genes for at least one of the TroA subfamilies, NRAMP proteins, or ZIP family proteins (Fig. 1), and roughly half have copies of genes for multiple families. In contrast, SAR11 genomes have reduced diversity of Mn and Zn transport systems compared to Roseobacter genomes. The only *troA* subfamily present in SAR11 appears to be the gene for the high-affinity Zn transporter *znuA*, present in 50% of the genomes (Fig. 1). In these genomes, the synteny of the *znuA* uptake system is highly conserved, with all regions containing a Fur-family protein gene as well as genes for the additional ABCT components. The putative ZnuA amino acid sequences have approximately 43% sequence identity to curated ZnuA sequences in UniProt, supporting their assignment as Zn transporters.

Nickel and cobalt transporters vary between Roseobacter and SAR11 genomes (Fig. 1). Ninety percent of Roseobacter genomes contain genes homologous to *hupE* and *ureJ*, which encode nickel transporters, while only *Citreicella* sp. strain SE45 has the well-characterized nickel transporter gene *nikA*. Forty-five percent of SAR11 genomes contain putative *hupE* and *ureJ* systems. In both SAR11 and Roseobacter, *hupE* and *ureJ* gene neighborhoods are highly variable, although in both groups, many *hupE* and *ureJ* genes are colocalized with ABCTs, suggesting a role in transport. Seventy-six percent of Roseobacter genomes have at least one of the *cbiMQ*, *cbtAB*, or *corA* cobalt uptake systems, and half have more than one kind of cobalt transporter. The most common Roseobacter cobalt transporter gene is *corA*, found in half of the genomes. In contrast, CorA is the only potential cobalt ion transporter in SAR11, and its gene is present in only the earliest-diverging genome, HIMB59. Ninety-eight percent of Roseobacter genomes contain *copA* systems, while 50% contain *copZ* (Fig. 1). In contrast, SAR11 appears to have completely eschewed the use of *copA*, but 54% of genomes contain *copZ* metallochaperone genes.

### Phylogenetic conservation of metal uptake genes.

We used the τ_*D*_ statistic (trait depth) from the consenTRAIT algorithm (69) and Fritz and Purvis' *D* for phylogenetic dispersion (68) to predict the extent to which Roseobacter and SAR11 phylogeny explains the distribution of metal uptake categories in each genome. The use of two independent approaches also allowed us to qualitatively assess the degree of uncertainty for our phylogenetic conclusions. When τ_*D*_ is large, a metal uptake trait will be shared among members of deeply branching clades, suggesting that these traits are consistently passed on to daughter lineages. When τ_*D*_ is small, the trait will more likely be found in small, dispersed clades in a phylogeny. Such dispersion could suggest that the trait is evolutionarily labile, having been lost and gained multiple times during the evolutionary trajectory of a microbial lineage. In the roseobacters, 20% of metal uptake traits exhibit τ_*D*_ values significantly greater than those observed from random permutation, while in SAR11, 33% are nonrandomly distributed (Table 1; also, see Fig. S2 in the supplemental material). This indicates that the remaining metal uptake traits are indistinguishable from patterns of random or convergent evolution based on the current number of genomes surveyed in each group. Generally, there is poor agreement between τ_*D*_ and the independent Fritz and Purvis' *D* metric as to which traits are nonrandomly distributed. However, both metrics estimate that a similar proportion (20% to 35%) of traits are nonrandomly distributed. Trait depth values for trace metal transport categories shared between Roseobacter and SAR11 are directly comparable because their phylogenies are based on the same protein families (Table 1; also, see Fig. S2 in the supplemental material). Four of the six shared categories have significantly greater trait depths in SAR11 than Roseobacter.

**TABLE 1:** Trait depth of metal uptake genes with significant phylogenetic signal

Gene/protein^*a*^	Metal(s) or process	τ_D_^*b*^			
		Roseobacter		SAR11	
		Mean	SD	Mean	SD
*afuA*	Fe^3+^	***0.0638***	***0.0040***		
PBP2 Fbp-like 1*	Fe^3+^	**0.0414**	**0.0020**	*0.0407*	*0.0017*
PBP2 FutA1-like***	Fe^3+^	***0.0565***	***0.0068***	*0.1781*	*0.0091*
*hutB*	Heme	*0.0274*	*0.0015*		
TBDT MCL2	Catecholate	*0.0331*	*0.0015*		
*fatB*	Catecholate	*0.0363*	*0.0016*		
TBDT MCL3	Hydroxamate	*0.0355*	*0.0015*		
*fhuD*	Hydroxamate	**0.0462**	**0.0021**		
SIP	Siderophore utilization	**0.0476**	**0.0021**		
ZIP	Fe^2+^, Zn^2+^	*0.0386*	*0.0028*		
znuA***	Zn^2+^	*0.0271*	*0.0024*	**0.0562**	**0.0021**
*psaA*	Mn^2+^	***0.0423***	***0.0056***		
*troA-a*	Mn^2+^, Zn^2+^	*0.0263*	*0.0014*		
*cbtA*	Co^2+^	*0.0307*	*0.0018*		
*cbtB*	Co^2+^	*0.0346*	*0.0024*		
corA***	Co^2+^, Ni^2+^, Mg^2+^	*0.0400*	*0.0025*	**0.2785**	**0.0127**
*btuF*	Co^2+^ as vitamin B_12_			**0.0809**	**0.0046**
copZ***	Cu^+/2+^, other heavy metals	*0.0268*	*0.0013*	*0.0070*	*0.0010*
TBDT MCL5	Unknown/multiple	**0.0441**	**0.0025**		
Total TBDT***	Multiple	***0.0493***	***0.0039***	*0.0652*	*0.0032*

a Single asterisks indicate a small Cohen's *d* effect size (*d* < 0.5) between Roseobacter and SAR11 means, and three asterisks indicate a large effect size (*d* > 1).

b Values in bold denote nonrandom phylogenetic distribution, as assessed by the consenTRAIT algorithm (*P* < 0.1), and those in italics denote nonrandom phylogenetic distribution from the independent *D* metric for phylogenetic dispersion of Fritz and Purvis (68) [*P*(*D*)_random_ < 0.05]. For all transporters shared by Roseobacter and SAR11, differences in trait depth for metal uptake traits are significant (Student's *t* test, *P* < 0.05).

### Relationships between patterns of metal uptake genes, habitat, and genome features.

**FIG 2:**
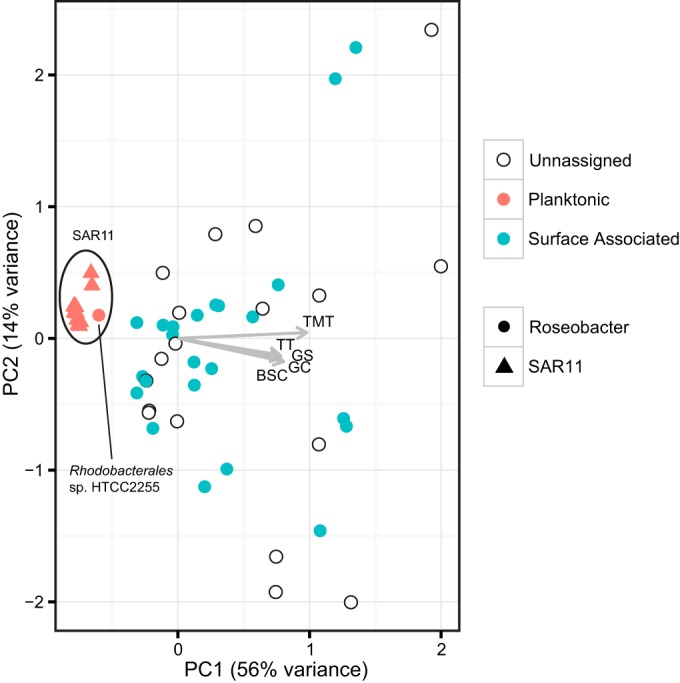
Ordination plot of a principal components analysis (PCA) based on the diversity and abundance of trace metal transporters in Roseobacter (circles) and SAR11 (triangles) genomes, as presented in Fig. 1. The 42 Roseobacter genomes and 22 SAR11 genomes are plotted with respect to whether the organism has been observed to be surface associated (blue) or planktonic (red) or there is insufficient data to assign a lifestyle (white). Arrows represent fitted vectors of continuous associated variables (genome features) and show the direction of the increasing gradient. Arrow length is proportional to the correlation between the variable and ordination. TMT, total number of metal transporters per genome (*R*^2^ = 0.96, *P* = 0.001); TT, total number of transporters per genome (*R*^2^ = 0.71, *P* < 0.001); BSC, total number of predicted biosynthetic gene clusters per genome (*R*^2^ = 0.63, *P* < 0.001); GC, GC content per genome (*R*^2^ = 0.67, *P* < 0.001); GS, total number of predicted genes per genome (*R*^2^ = 0.63, *P* < 0.001).

As metals are essential cofactors in a variety of ecologically relevant metabolic processes, we tested for associations between the abundance of metal transport systems, microbial habitat, and genome features. A principal components analysis (PCA) based on diversity and abundance of trace metal transporters indicates that SAR11 genomes cluster tightly in ordination space, while Roseobacter genomes are highly dispersed (Fig. 2). The differences in ordination dispersion between Roseobacter and SAR11 are statistically significant (see the results and methods in the supplemental material). Factors corresponding to ocean basin of isolation and coastal versus pelagic isolation are not significantly associated with the PCA ordination. We classified genomes as having a planktonic or surface-associated lifestyle based on whether a strain was described as associating with particles experimentally (see Data Set S6 in the supplemental material) (72) or was isolated from a biotic or abiotic surface. Strains for which lifestyle data could not be clearly determined were omitted from statistical analyses. As a result, surface-associated and planktonic factors are significantly associated (*R*^2^ = 0.33, *P* < 0.001) with the reduced PCA ordination (Fig. 2). The total number of metal transporters, the total number of transporters in each genome mapping to the Transporter Classification Database (TCDB) (73), the predicted number of biosynthetic gene clusters per genome, the GC content, and number of genes per genome are all strongly correlated with the ordination. The number of predicted laterally transferred genes is not significantly correlated.

**FIG 3:**
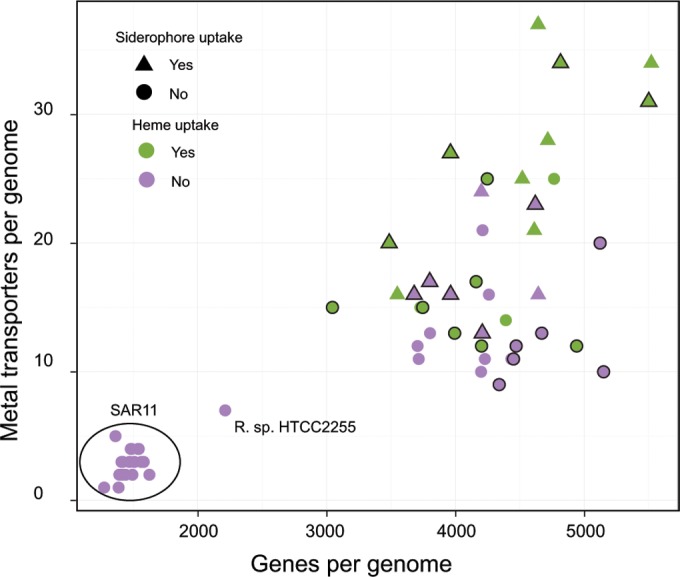
Scatter plot of metal transporters per genome plotted against genes per genome. Each point represents one genome and is colored according to the genomic potential for heme uptake and shaped according to the genomic potential for siderophore uptake. Points with a black outline represent organisms that have been experimentally confirmed to be particle associated or were isolated from an abiotic or biotic surface. The SAR11 genomes and the HTCC225 genome are the only genomes from organisms with confirmed planktonic lifestyles. Spearman's rank correlation coefficient (ρ) values were as follows: for SAR11 genomes (*n* = 22), ρ = 0.37 (*P* > 0.05); for Roseobacter genomes (*n* = 42), ρ= 0.31 (*P* < 0.05); for combined genomes (*n* = 64), ρ= 0.78 (*P* < 10^−10^).

In the combined Roseobacter-SAR11 data set (see Fig. S3A in the supplemental material) and the data set for Roseobacter alone (see Fig. S3B in the supplemental material), there is a significant positive correlation between metal transporter abundance and transporters predicted to be in the same pathway based on synteny and sequence homology. Ultimately, metal uptake pathways for small, defined iron complexes (siderophores and heme) are best correlated with genome features such as increasing genome size, increasing number of total transporters per genome, and increasing metal transporters per genome. Indeed, it does generally appear that siderophore and heme uptake are largely biased toward the largest genomes with the most transporters, but they are also unevenly distributed in Roseobacter (Fig. 3). The strongest positive correlations for the combined and individual lineages are between the total number of transporters per genome and the genome size (see Fig. S4A in the supplemental material). However, the number of metal transporters per genome has the poorest correlation with genome size (see Fig. S4C in the supplemental material) and marginally better correlation with the total number of transporters (see Fig. S4D in the supplemental material).

## DISCUSSION

Consistent with streamlining theory (37), the background-adapted SAR11 lineage has relatively few trace metal transporters and an apparently limited regulatory capacity for Fe uptake. In contrast, the genomes of the mostly patch-adapted Roseobacter strains investigated here have multiple diverse pathways for the acquisition of both organically complexed and inorganic metals. This suggests that roseobacters are able to adapt to and occupy a range of trace metal niches in the marine environment and that the availability of trace metal resources may influence Roseobacter genome diversification. The variable inventories of trace metal transporters in Roseobacter and SAR11 may ultimately reflect variable metabolic demands for metals as enzymatic cofactors. However, no studies, to our knowledge, have specifically examined metal quotas for SAR11 and Roseobacter strains. A few Roseobacter genomes (for example, HTCC2255) appear closer in gene content to background-adapted SAR11 genomes (44, 50), suggesting that trace metal streamlining is also a valuable ecological strategy for some roseobacters. Indeed, background adaptation is probably a more prominent strategy in the Roseobacter lineage than currently available isolates would suggest (44). Thus, the results presented here for Roseobacter are likely biased toward the portions of the lineage that are patch adapted.

### Similarities between SAR11 and Roseobacter genomes.

Fe^3+^ ABCTs are the most abundant Fe transporter identified in this study, suggesting that free Fe^3+^ is the most common form of Fe in the periplasm in both Roseobacter and SAR11. All investigated genomes on either end of the background-adapted–patch-adapted spectrum contain ABCT transporters for Fe^3+^ uptake, with the exception of the genomes of the SAR11 strains AAA240-E13 and HIMB058, although both these genomes may be missing these transporters due to genome incompleteness. Nevertheless, it appears that ABCT uptake is likely the default mechanism for Fe^3+^ uptake across the bacterial inner membrane for both the SAR11 and Roseobacter lineages and may be an essential system. Fe^2+^ is often at very low concentrations in the marine environment, although at times it can accumulate to significant proportions of the total Fe pool (74). However, Fe^2+^ transporters are rare in both SAR11 and Roseobacter.

### Differences between SAR11 and Roseobacter genomes.

Roseobacter and SAR11 genomes are mostly different with respect to trace metal transporter inventory. Even though Fe^3+^ ABCTs are basically present in all genomes, 30 out of 42 Roseobacter genomes have multiple copies of Fe^3+^ ABCTs from at least two different domain families (TrichCH4B has five), while only four of the 22 SAR11 genomes have multiple copies. This suggests a nuanced distinction between patch-adapted Roseobacter and background-adapted SAR11 that may be reflective of Fe niches. For example, the existence of multiple versions of substrate transporters has been invoked to explain multiphasic kinetics for glucose uptake in bacterial isolates and natural assemblages (75, 76).

The Fur protein family has a large diversity of metal selectivity, making it challenging to assign specific metal cofactors using sequence homology alone (77). Regardless, Roseobacter genomes generally have multiple copies of Fur-like regulatory proteins, and many of their iron uptake genes are downstream of Fur box regulatory motifs. Past work suggests that Fur proteins are present only in the genomes of SAR11 clade Ia members (49), but our results indicate that all SAR11 genomes have some form of a metal-dependent transcription factor. It appears that SAR11 has greatly downsized its metal-dependent regulatory networks compared with patch-adapted roseobacters, which is consistent with prior observations of its overall regulatory complexity (37). This suggests that patch-adapted roseobacters have the ability to sense and react to a wide variety of trace metal forms, while the background-adapted SAR11 organisms may respond to only a limited portion of the overall trace metal chemical diversity in seawater.

Many of the Fe-binding ligands identified from cultured marine bacteria (78) have been siderophores, low-molecular-weight and high-affinity Fe^3+^-chelating agents secreted by some bacteria explicitly for the purpose of chelating Fe. Siderophores have also been detected in bulk seawater, are predicted to shape bacterial social interactions (79), may reflect degrees of habitat structure (80), and are hypothesized to comprise a significant component of the marine strong iron-binding ligand pool (81). Therefore, it is plausible that siderophore and perhaps other undiscovered strongly bound iron complexes are important iron sources for some marine bacteria. Forty percent of roseobacters can probably acquire at least one type of siderophore or other small-molecule Fe chelator, and ∼10% have the potential to produce siderophores. Indeed, both Phaeobacter strains included in this study have been shown to produce siderophores in culture (82). The discrepancy between proportions of siderophore producers and those with the potential for uptake may reflect public goods dynamics observed in other copiotrophic marine bacteria (79). In contrast, no SAR11 genomes have either capability, and siderophores have been used to experimentally Fe limit “Candidatus Pelagibacter ubique” HTCC1062 in culture (83). Here, our focus was on direct transport of siderophore complexes, and this study was not meant to address the possibility of extracellular processing of organically bound Fe into bioavailable forms, although this is certainly an important yet understudied possibility. Our results suggest that direct uptake of small intact organic-Fe complexes via TBDTs is an important uptake strategy for some, but not all, roseobacters, while it appears to be an expendable strategy for the background-adapted SAR11 organisms.

Heme *b*, another form of organic Fe, is a dynamic and significant component of the marine Fe cycle (22). Like siderophores, no heme uptake systems were detected in SAR11 genomes. Interestingly, about equal proportions of Roseobacter genomes from this study have heme and siderophore uptake systems (45% and 40%, respectively), and nine strains have only a single TBDT, which is specific for heme. The Roseobacter heme uptake gene locus is also highly conserved with respect to synteny and TBDT sequence similarity, suggesting a tightly controlled evolution of this gene cluster.

The proportion of heme uptake systems in Roseobacter identified here is consistent with what has been described earlier (84), but it is interesting that the prevalence of heme uptake is roughly equivalent to that of siderophore uptake. This is significant in that siderophores are the dominantly researched Fe-ligand complex in marine systems. In the case of the Roseobacter clade, it appears that heme may be equally important as siderophores, which may be due to the frequent association of Roseobacter with hemoprotein-rich phytoplankton (22, 46). Supporting this hypothesis, many roseobacters with heme uptake systems were isolated from phytoplankton or are known to associate with other organisms (38).

How do background-adapted members of SAR11, one of the most abundant microbial lineages on earth, manage to satisfy their iron requirements by apparently utilizing only inorganic Fe^3+^, the scarcest form of oxidized iron in the oceans (4)? One hypothesis is that SAR11 cells directly modify refractory extracellular dissolved organic, colloidal, or particulate Fe species into usable forms. Another hypothesis is that SAR11 relies on the activity of external agents in microbial ecosystems to produce enough labile Fe for its survival, analogous to how Prochlorococcus cells appear to rely on the activity of microbial community members for hydrogen peroxide oxidation (85). Background-adapted organisms like SAR11 often coexist in food webs strongly controlled by micrograzers and viruses, in which regular biomass turnover may produce significant and regularly occurring labile Fe sources. For example, if there is a strong diel structuring of Prochlorococcus mortality, as has been observed previously (86), cooccurring heterotrophic bacteria may in turn synchronize the expression of trace metal transporters to daily periods of increased Prochlorococcus lysis. In support of this idea, many Roseobacter, SAR11, and SAR116 transporter transcripts displayed strong diel periodicity in a recent field study (87). Ultimately, our results indicate that SAR11 cells do not have the ability to directly transport intact organic iron complexes, suggesting that they utilize Fe ions or very small charged complexes which are moved through the outer membrane probably by passive transport. SAR11 appears to produce large amounts of ABCT solute binding proteins under Fe stress (83), and solute binding proteins are highly abundant in natural populations (88). It may be that highly expressed solute-binding proteins efficiently intercept all periplasmic Fe^3+^ and drive a gradient inward toward the cytoplasm and/or that unknown cell surface binding proteins operate to locally concentrate Fe at the outer membrane.

Mn, Zn, Ni, Co, and Cu are cofactors in many biogeochemically significant metabolic pathways and are important micronutrients for marine heterotrophic bacteria. Most Roseobacter genomes contain dedicated inner-membrane transporters for transition metals other than Fe, and many have apparently redundant systems for some metals. For example, two *troA* superfamily substrate-binding proteins identified in this analysis from Ruegeria sp. strain TM1040 and Roseobacter sp. strain AzwK-3b have been shown to be highly expressed under Mn-limiting conditions (89). As with organic Fe transporters, Mn, Zn, Ni, Co, and Cu transporters were largely absent in background-adapted SAR11 genomes. However, an intriguing exception suggests that some metal uptake traits have escaped the purging effect of genome streamlining in SAR11. Early-diverging SAR11 lineages and some SAR11 group Ia members appear to occupy niches where high-affinity zinc uptake (*znuA*) is useful, while roseobacters appear to have mostly rejected this trait. Although the SAR11 *znuA* sequences are quite similar to characterized *znuA* proteins from model bacteria, there is a possibility that they are involved in the uptake of other metals. Physiological experiments are needed to confirm substrate specificity, but based on the level of sequence similarity, we anticipate that our bioinformatic predictions here are robust. Currently, it is unknown whether the presence of *znuA* in these SAR11 strains represents greater absolute Zn requirements relative to other trace metals, reduced Zn concentrations in specific niches, or some other factor. Genomes with *znuA* do not contain more annotated Zn-binding domains than other SAR11 genomes, nor are they significantly connected by isolation source as it is defined in this study. However, it is intriguing that most SAR11 strains isolated from pelagic surface waters have *znuA*, which may be related to the extremely low concentrations of Zn and other metals in the pelagic surface ocean.

### Phylogenetic signal in metal uptake traits.

This study contextualizes trace metal transporters within a phylogenetic framework in marine microbes in order to explore combined patterns of heritability, gene loss, and lateral transfer. Our results indicate that the majority of metal uptake traits are not significantly associated with phylogeny in either SAR11 or Roseobacter, and it appears that in both groups trace metal niche adaptation has occurred through evolutionary mechanisms indistinguishable from stochastic processes. A higher percentage of metal transporters do appear to be nonrandomly distributed in SAR11 than Roseobacter potentially suggesting an overall greater role for vertical heritability in SAR11. Furthermore, certain metal uptake traits shared between groups cluster at significantly different clade depths, which implies that distributions of these families across the SAR11 lineage are more likely to be fixed across fine-scale phylogenetic diversity than they are in the Roseobacter lineage.

Recent surveys of carbon utilization traits (69) and extracellular enzymes (90) using the consenTRAIT metric suggest that these traits mostly exhibit nonrandom phylogenetic distribution despite their generally shallow clade depth. We show here that most metal uptake traits in both Roseobacter and SAR11 also have shallow clade depths (small τ_*D*_) but appear to be randomly associated with their reference phylogenies. We interpret these results as potentially reflecting differing degrees of selective pressure with respect to specific metals in the Roseobacter and SAR11 lineages. Microbial niche exploration, changing trace metal availability in existing niches, or altered absolute metal requirements may have resulted in gene-specific selective sweeps in both patch-adapted and background-adapted Roseobacter and SAR11 lineages. These processes may also have resulted in selective loss of capabilities as well. Although the τ_*D*_ and *D* metrics cannot distinguish between lateral gene transfer or selective gene loss events, the result is that phylogenetically similar strains within each lineage have strongly differentiated with respect to trace metal uptake genes. We did not explore mechanisms of selective gene gain versus loss in Roseobacter and SAR11 here, but our results are consistent with the hypothesis that marine trace metal resource availability is a selective pressure influencing genome content in both patch-adapted and background-adapted genomes from the Roseobacter and SAR11 lineages.

### Metal transporters, habitat, and genome features.

The four factors most strongly correlated with the metal uptake ordination are the number of metal transporters per genome, the GC content of each genome, the number of total transporters per genome, and genome size. An inspection of individual pairwise correlations shows that genome size is strongly positively correlated with many specific uptake pathways, but many of these specific correlations are lost when only Roseobacter genomes are included. In general, it appears that smaller Roseobacter genomes have fewer total metal transporters, but metal transporters do not neatly scale with genome size in many other cases. Octadecabacter arcticus 238 has the third largest genome of all roseobacters, but it has no TBDTs and appears to have systems only for Fe^3+^ and Mn^2+^ uptake. In contrast, half of the 10 smallest Roseobacter genomes have transporters for heme and siderophore-like complexes.

Habitat categories (e.g., coastal, pelagic, and ocean basin isolation) as defined in this study are not correlated with the trace metal uptake inventory in either Roseobacter or SAR11. However, significant correlation exists when only strains with confirmed particle attachment lifestyles are considered. Supporting these results, recent meta-omic studies (91–93) have demonstrated that prefilter size (particle fractions) is a better predictor of differences in community structure, metabolic capability, and transcriptional activity than both depth and geographical variability. However, many uncultured roseobacters are predicted to be background adapted and may not associate with particles at all (44, 45), and it is unknown whether genomes of uncultured roseobacters contain genes for organic Fe uptake systems. Therefore, it is possible that trace metal uptake inventory may not overlap neatly with a particle-adapted lifestyle in the Roseobacter lineage due to a culturing bias and/or incomplete lifestyle assignments. HTCC2255, the only background-adapted member of the Roseobacter whose genome is included here, is thought to be only free living (50) and is similar in trace metal transporter inventory to SAR11. More work is needed to obtain full genome sequences of background-adapted roseobacters and to understand the potential for particle attachment in both background and patch-adapted strains.

Our results indicate that the genomes of both patch-adapted and background-adapted organisms included in this study exist along spectrums of trace metal acquisition capability. At one extreme, genomes contain multiple and apparently redundant pathways for the uptake of most metals, while at the other end they lack many transport families. In patch-adapted roseobacters, the presence/absence of any particular metal uptake pathway is not predictable from genome size alone, and only a small subset of all metal uptake genes are present in all genomes. This also indicates that the presence of any one metal acquisition pathway, such as siderophore or heme uptake, is not representative of the capabilities of the Roseobacter lineage as a whole. Even though the genomes of background-adapted organisms included in this work have reduced metal uptake capabilities compared with those from patch-adapted organisms, they also appear to have a surprising degree of variability, for example, the *hupE* and *ureJ* family and the patchy distribution of *znuA*. We interpret this as reflecting degrees of trace metal niche differentiation, whereby marine trace metal concentrations and chemical speciation influence genomic content at fine levels of phylogenetic differentiation in both background strategists and patch strategists. Thus, trace metal niches should be considered an important factor in shaping the genomic content of marine heterotrophic bacteria and should be considered when microbial roles in broader marine ecosystems and biogeochemical cycles are examined.

## Supplementary Material

Supplemental material
