# Supplementary material for: Trace Metal Acquisition by Marine Heterotrophic Bacterioplankton with Contrasting Trophic Strategies
Source: Appl Environ Microbiol. 2016 Feb 19;82(5):1613–24. doi: 10.1128/AEM.03128-15 (PMC4771312; doi:10.1128/AEM.03128-15)
Supplement: Supplemental material [file supp_82_5_1613__index.html]

Supplemental material 

# Trace Metal Acquisition by Marine Heterotrophic Bacterioplankton with Contrasting Trophic Strategies

## Supplemental material

**Files in this Data Supplement:**

- Supplemental file 5 -

  Supplemental results and discussion, supplemental methods, metal transport components and their frequency (Table S1), TBDT Markov clusters and their occurrence in *Roseobacter* genomes (Table S2), examples of TRAP transporters in genome neighborhoods with genes involved in siderophore biosynthesis or siderophore-like compound uptake (Fig. S1), box plots (Fig. S2), network visualization (Fig. S3), and scatter plots (Fig. S4).

  PDF, 1023K
- Supplemental file 1 -

  Genome features, genome completion, environmental characteristics, and transporter abundance (Data Set S1).

  XLSX, 26K
- Supplemental file 2 -

  COGs used for phylogenetic tree construction (Data Set S4).

  XLSX, 11K
- Supplemental file 3 -

  TonB-dependent receptor genome neighborhoods and Markov clustering parameters (Data Set S5).

  XLSX, 20K
- Supplemental file 4 -

  Particle-associated versus free-living lifestyle assignments and references used to make assignments (Data Set S6).

  XLSX, 11K
- Supplemental file 6 -

  Predicted Fur boxes (Data Set S3).

  XLSX, 21K
- Supplemental file 7 -

  Predicted Fur boxes (Data Set S3).

  XLSX, 19K
