# Supplementary material for: Trace Metal Acquisition by Marine Heterotrophic Bacterioplankton with Contrasting Trophic Strategies
Source: Appl Environ Microbiol. 2016 Feb 19;82(5):1613–24. doi: 10.1128/AEM.03128-15 (PMC4771312; doi:10.1128/AEM.03128-15)
Supplement: Supplemental material [file AEM.03128-15_zam999116977so1.pdf]

Supplemental text for:

Hogle, SL, Thrash JC, Dupont CL, Barbeau KA. Trace metal acquisition by marine heterotrophic bacterioplankton with contrasting trophic strategies. ***Applied and Environmental Microbiology***

In addition to being hosted at the *Applied and Environmental Microbiology* website, all supplemental files, additional code, and datasets are available at:

http://dx.doi.org/10.6084/m9.figshare.1533034

Supplemental file descriptions:

- Supplemental_text.pdf - Supplemental results and methods
- Dataset1.xlsx - Genome features, Genome completion, environmental characteristics, transporter abundance
- Dataset2.xlsx - CDD/PFAM/COGs used to identify metal transporters
- Dataset3.xlsx - Predicted FUR boxes
- Dataset4.xlsx - COGs for Tree Construction
- Dataset5.xlsx - TBDT neighborhoods, and Markov Clustering parameters
- Dataset6.xlsx - Particle associated versus free-living lifestyle assignments and references used to make assignments

Supplemental Results and Discussion:

Genome features: The strains in this study were isolated from a variety of ocean basins, both coastal and pelagic waters, and from particles or surfaces and bulk seawater. 56 of the genomes are either closed or in permanent draft status with the remaining 8 in draft status. The genomes range from 1 to 339 scaffolds and are estimated to be between 93.8% to 100% complete. The genome sizes range from 1.11 Mbp (*Candidatus Pelagibacter ubique* HIMB05) to 5.52 Mbp (*Citreicella* sp. SE45) and code for between 1269 (*Candidatus Pelagibacter ubique* HIMB058) to 5519 (*Pelagibaca bermudensis* HTCC2601) genes. GC content ranges from 29% (*Candidatus Pelagibacter* sp. HTCC7211) to 70% (*Oceanicola granulosus* HTCC2516). The single cell SAR11 AAA240-E13 genome is least complete (93.8%) but is included here to increase the phylogenetic diversity of SAR11 genomes in our analyses.

Unknown tonB dependent transporters and other secondary transporters - Implications for atypical metal uptake: TonB dependent transporters (TBDTs) have a broad range of substrates, but

interestingly, 77% of Roseobacter TBDTs appear to be involved in the uptake of small Fe complexes based on gene neighborhood, sequence similarity, and predicted FUR regulation. This is in contrast to TBDTs in other marine groups such as SAR86 (1) and the phylum *Bacteroidetes* (2), which appear to be mostly dedicated to the uptake of high molecular weight carbon compounds. TBDTs in clusters MCL6 and MCLnull were the most difficult to assign substrates from gene context and sequence similarity, but were located next to substrate binding proteins for peptide and sugar transport as well as non-specific metal transport. Interestingly, TRipartite ATP-independent Periplasmic (TRAP) transporters are present within two putative siderophore uptake clusters (Fig. S1). TRAP transporters utilize a substrate binding protein to shuttle substrates in the periplasm, but the substrate is moved through the inner membrane by the cotransport of a counter ion (usually Na^+ or H^+) in the opposite direction (3), rather than the hydrolysis of ATP. The use of TRAP transporters in conjunction with TBDTs (particularly siderophore transporters) has thus far been unexplored (4). The colocalization of TRAP transporters with seven different putative Fe TBDTs and siderophore biosynthesis genes (Fig. S1) is unique, and to our knowledge has not been reported before. It has been postulated that using two counter Na^+ ions would impart a significant energy savings compared to the use of an ATP binding cassette transporter (ABCT) system (5). In the marine environment where substrates are highly dilute, the energy savings of utilizing TRAP transporters over ABCTs may be significant, especially when used in concert with ion gradients generated by proteorhodopsins (6). Every Roseobacter genome contained at least eight different substrate binding protein components of TRAP transporters with *Citreicella* sp. SE45 containing over 40 different homologs. TRAP uptake is constrained by the presence of at least one carboxylic acid group in the substrate (3). Carboxylic acid groups are frequently involved in the chelation of metal species (7) making metal-ligand complexes a potential substrate for TRAP transporters. The identification of TRAP transporters next to siderophore biosynthesis genes and siderophore TBDTs implies a previously unrecognized role in Fe chelate transport for the TRAP family.

Phylogenetic conservation of metal uptake genes

Genes coding for microbial cellular functions can be differentially conserved with respect to a phylogeny derived from a universal and conserved reference gene such as the 16S rRNA gene. The degree of correspondence of a particular trait with an organism's phylogeny can be thought of in terms of phylogenetic trait depth. We used the τ_D statistic (trait depth) from the consenTRAIT algorithm (8) and Fritz and Purvis' D for phylogenetic dispersion (9) to predict the extent to which *Roseobacter* and SAR11 phylogeny (Fig. 1) explains the distribution of metal uptake categories in each genome. The use of two independent approaches also allowed us to qualitatively assess the degree of uncertainty for our phylogenetic conclusions. The τ_D statistic is a continuous metric that corresponds to the mean branch length between root nodes and leaves in a phylogenetic tree where 90% of the leaves have a particular trait. Unlike D, trait depth is not normalized to phylogeny size and represents direct phylogenetic distances with larger values indicating deeper clades. Fritz and Purvis' D is calculated by the sum of changes in nodal values of a binary trait along edges in a phylogeny. The metric is robust for phylogeny sizes down to approximately 25 leaves and where trait prevalence is greater than 0.2 (see methods and supplementary materials for details).

We hypothesized that patch-adapted and background-adapted genomes would have differing signatures of heritability for trace metal uptake due to the constraints of genome size. Whereas larger genomes contain more genetic material upon which processes of recombination and lateral transfer can operate, background-adapted genomes would have streamlined to such an extent that essential trace metal uptake pathways would have been largely phylogenetically fixed across a lineage. 57% of the estimates of phylogenetic dispersion in *Roseobacter* are less than 0.5, but only 35% of traits have probabilities of matching a stochastic distribution of less than 5% ($P(\mathrm{D})_{\mathrm{random}} < 0.05$) and have prevalence values greater than 0.2 (*Prevalence* > 0.2). In SAR11, 55% of D values are less than 0.5, but only 33% of traits have *Prevalence* > 0.2 and $P(\mathrm{D})_{\mathrm{random}} < 0.05$ (Table 1, Fig. S2). We believe our results reflect an

authentic lack of phylogenetic signal in trace metal uptake traits because two independent metrics generally agree in terms of proportions of phylogenetic signal in both patch and background-adapted genomes. We interpret these results as reflecting differing degrees of selective pressure with respect to specific metals in the *Roseobacter* and SAR11 lineages. Microbial niche exploration, changing trace metal availability in existing niches, or altered absolute metal requirements may have resulted in gene-specific selective sweeps in both patch-adapted and background-adapted lineages.

Ordination: The multivariate homogeneity of group dispersions (Fig. 2) for *Roseobacter* has a greater average distance to the median (4.227) compared to SAR11 (1.087) and this difference is significant ($P < 0.001$). The larger dispersion value indicates that *Roseobacter* genomes have greater within-group variability in uptake capabilities than SAR11. For example, the *Roseobacter* HTCC2255 genome is more similar to a SAR11 genome (distance to SAR11 mean centroid of 1.16) than it is to the average *Roseobacter* genome (distance to *Roseobacter* mean centroid of 3.39). Multivariate homogeneity determined using Bray-Curtis dissimilarity (with non-metric multidimensional scaling) also indicates that the *Roseobacter* dispersion is significantly larger than SAR11 demonstrating the robustness of the trend.

Correlations between metal transporters and genome features: In the combined *Roseobacter* and SAR11 dataset, many of the correlations between genome features and metal transport categories are driven by the inclusion of SAR11. For example the strong negative correlation between *znuA* and many genome features is a result of its prevalence in SAR11 genomes. In *Roseobacter* (Fig. S3B), the number of metal transporters per genome, the total transporters per genome, and GC content are strongly positively correlated with TBDT categories. The strongest correlations when considering both SAR11 and Roseobacter genomes (Fig. S3A) are between genome features (grey diamonds) and

nonspecific metals (blue) as well as between transporters predicted to be in the same uptake pathway (eg. TBDT MCL1, hemS, and hutB). The large number of *troA* superfamily proteins, siderophore transporters, and heme transporters in Roseobacter are positively correlated with genome features such as increasing genome size and increasing number of total transporters. Ultimately, metal pathways related to uptake of small defined iron complexes (siderophores and heme) are best correlated with genome features such as increasing genome size and increasing number of total transporters per genome. A previous study reported a statistically significant negative correlation between high-affinity Fe^{2+} transporters and Fe^{3+} transporters suggesting that marine bacteria tend to rely on either *feoB* or Fe^{3+} ABCTs but not both (10). We do not see this negative correlation in our results, neither do we observe a positive correlation between ZIP divalent metal transporters and Fe^{3+} ABCTs as was also reported earlier (10). However, the previous study included genomes from many different taxonomic groups, and those trends may have been driven mostly by specific bacterial taxa not included in our study. In the case of the *Alphaproteobacteria* genomes examined here it appears that neither Fe^{3+} or Fe^{2+} uptake capabilities are exclusive of one another and indeed many strains have the capabilities for both reduced and oxidized Fe uptake. Interestingly, the number of genes per genome predicted to be laterally transferred was positively correlated with genes related to siderophore biosynthesis and processing, suggesting a potential linkage between lateral gene transfer and the ability to synthesize siderophores.

The stark difference between the trace metal uptake inventory in SAR11 and *Roseobacter* generates a number of correlations that are not observed when examining either group individually. Effectively no significant correlations exist between genome features and metal uptake pathways in SAR11 genomes because they are lacking most transporters observed in this study. When considering only Roseobacter genomes (Fig. S3 B), the total number of metal transporters per genome are correlated with 14 metal transport categories, most having to do with siderophore and heme uptake. The total number of

Transporter Classification Database (TCDB) (11) transporters per genome are correlated with four metal transport categories (MCL6, MCLnull, ZIP, and P_{1B} ATPases) as well as the total number of metal transporters. GC content is also correlated with the total number of transport genes per genome, ZIP transporters, and P_{1B} ATPases. Genome size, the number of predicted biosynthetic clusters, and the total number of genes with a functional prediction only correlated with with the TCDB count and no specific metal uptake pathway.

Uptake systems for Vitamin B_{12}: The single cell SAR11 genome AAA240-E13 contains a troA superfamily protein with strong similarity to the vitamin B_{12} solute binding protein, *btuF*. On a different scaffold, AAA240-E13 has a TBDT with strongest similarity to a vitamin B_{12} receptor *btuB*, which suggests this genome may encode the potential for vitamin B_{12} uptake. In addition, the genome of HIMB114 has a putative *btuB* vitamin B_{12} TBDT, but it is unclear whether these systems would be functional. SAR11 genomes lack B_{12} biosynthesis pathways, which supports the hypothesis that in some niches B_{12} is scarce enough to warrant a dedicated transport system in background-adapted microbes. However, neither SAR11 genome appears to have the energy transduction machinery necessary for TBDT function, casting doubt as to whether the TBDTs are actually functional. All SAR11 genomes are missing most of the genes required for vitamin B_{12} biosynthesis.

Siderophore uptake and public goods: Even though ~40% of Roseobacter genomes appear to have siderophore uptake systems, very few strains appear able to biosynthesize siderophores. This suggests that most Roseobacters with putative organic iron-complex TBDTs rely on siderophores or small Fe chelating ligands of similar structure that are not endogenously produced as secondary metabolites. This observation is consistent with a "public goods" dynamic that has been demonstrated for a large and deeply sampled grouping of particle associated *Vibrio* strains (12). In this case, siderophore

“cheaters” found on large particles do not contribute to the community pool of siderophores and are able to access the public goods generated by siderophore producers (12). Marine particles are often rich in roseobacters (13, 14), and these same public goods dynamics apparent in *Vibrio* communities may have driven the evolution of siderophore biosynthesis and uptake capabilities in Roseobacter communities. The apparent lack of siderophore biosynthesis in the *Roseobacter* clade may be due to an oversampling of siderophore cheater genomes. An alternative explanation is that putative siderophore uptake genes may in fact be targeted to unknown strong marine ligands with similar chemical moieties to siderophores

Supplemental Methods:

Genomic sequence data and genome classification schemes: Genome size, total gene counts, GC content, the number of genes with a functional prediction, the number of genes assignable to the Transporter Classification Database, the estimated number of genes predicted to be horizontally transferred, and the estimated number of biosynthetic clusters per genome were obtained from IMG database annotations, and are available in Dataset1. Isolation data (geographical and lifestyle) for bacterial strains used in this study are compiled from the primary literature. The specific references used are available in Dataset6. If isolation data could not be reliably determined from the literature then these data were omitted from subsequent statistical analyses.

Identification of metal transporters, biosynthesis genes, and metal-dependent enzymes: The NCBI Conserved Domain Database (COG/Pfam/CDD/TIGRFam) was used to classify protein sequences using local database searches with either rpsblast (15) or HMMER 3(16). To produce statistics comparable to web-based searches databases were formatted using default minimum word score and Pssm scale factors. All orthologs to metal transport families (Dataset2, Table S1) were identified by bi-

directional best hit to conserved domain database models and only "specific hits" to domain models were retained. Table S1 displays the domain families with respective metal specificities used in this study while supplemental Dataset2 lists domain accession identifiers. In the case of the *troA* superfamily, sequences matching the "TroA helical backbone superfamily domain" (cl00262) but not matching other specific subfamilies above a bitscore threshold were identified as hits to "*troA*-superfam" which we use to represent generic metal-interacting solute binding proteins. Nonribosomal peptide synthetase independent siderophore (NIS) biosynthesis capabilities were assigned if genomes contained NIS synthetases and NIS acetyl transferases. Nonribosomal peptide synthetase (NRPS) pathways synthesize many secondary metabolites including siderophores (17), so the presence of NRPS pathways cannot be simply attributed to siderophore production. To identify NRPS siderophore biosynthesis, all NRPS-like domains were identified then manually annotated using ferrichrome synthetase (18), enterbactin synthetase subunits E and F, the phosphopantetheinyl transferase component of enterobactin synthetase, and enterochelin esterase (17).The dataset containing detailed TBDT annotations is available in Dataset5. Abundances of multi-gene pathways or enzymes known to utilize a particular metal, for example [NiFe] hydrogenases or Vitamin B_{12} biosynthesis, were identified by orthology to KEGG through the IMG web service.

Markov Clustering of TonB Dependent Transporters (TBDT) and protein family enrichment: To classify TBDTs by sequence similarity, all TBDTs identified by rpsblast were bi-directionally evaluated using BLASTp using an Evalue cutoff of 1.00E-15. TBDTs were clustered using the MCL algorithm of the clusterMaker (19) plugin in Cytoscape. An edge weight conversion of -log(Evalue), edge weight cutoff of 1, a pruning threshold of 1.00E-015, 16 iterations, and a maximum residual value of 0.001 were used in the Markov Clustering. The number of clusters was explored using granularity parameters of 1.4, 2.0, 2.2, 2.5. Ultimately, a granularity parameter of 2.0 was chosen as it best partitioned TBDTs into clusters with neighboring genes of coherent function. The dataset containing

results of clustering using multiple granularity parameters is available in Dataset5. Substrate specificity for TonB Dependent Transporters (TBDT) was assigned by manually examining the genome neighborhood (10 genes upstream and downstream) for the presence siderophore SBPs, SIPs, siderophore biosynthesis genes, heme uptake genes, other metal uptake systems, and the Fur box DNA regulatory motif. Fisher's exact test was then used to test if protein families were enriched in TBDT gene neighborhoods (10 genes upstream and downstream of TBDT). Briefly, protein neighborhoods were collected from the uniprot database and COG/Pfam/CDD/TIGRFam domains existing in more than 20% of TBDT neighborhoods of a particular MCL cluster were enumerated inside and outside of the TBDT neighborhood and used in Fisher's exact test. *P* values from the Fisher's exact test were corrected using the Benjamini Hochberg method for controling the false discovery rate. Protein families were considered enriched if $P < 10^{-5}$ and the Odds Ratio was > 1. Results from the enrichment analysis are presented in Table S2.

Identification of Fur box DNA regulatory elements: The *Rhodobacterales* have previously been described to employ DNA regulatory motifs divergent from that of the canonical Fur box (20). As such, searches for the 19 DNA base pair inverted repeat consensus fur box (21) generally yielded poor matches in the Roseobacter genomes. To address this, a collection of 99 inverted repeat sections of the binding sequence for Fur proteins (Iron-Rhodo box) was obtained from a prior study utilizing ten different Roseobacter genomes (20). These sequences were used in a standard blastn search against the genomes of all 42 Roseobacter used in this study. This resulted in approximately 170 potential Roseobacter Fur boxes. These 170 sequences were collected and used in another blastn search against Roseobacter genomes using lenient search parameters including a match reward = +1, match penalty = -3, gapopen = +5, gapextend = +2, word size = 7, and no masking or filtering for low complexity. Regions were discarded if they contained less than 15 base pairs of exact similarity to an existing sequence or if they were not within an intergenic region. The regions of all new hits were manually

searched for candidate Fur-regulated genes based upon whether the gene's predicted function was related to iron uptake or homeostasis. If the newly identified Fur box was near a TBDT this information was used to update the potential substrate if necessary. In contrast, SAR11 genomes had readily identifiable Fur-box sequences based on a blastn search using the 15 basepair (7-1-7) inverted repeat identified in prior work(21). SAR11 genomes were searched with the 15 base pair motif using the same blastn parameters as described for the Roseobacters and candidate iron-regulated genes were identified. The identified Fur boxes, their genomic location, and the gene and/or operon that they putatively regulate are listed in Dataset3.

Phylogenetic tree inference: 26 of 28 composition-homogenous orthologous protein families (excluding COG0238 and COG0522) were identified in the 64 genomes (Dataset4). Amino acid sequences in each orthologous set were aligned using MUSCLE (22) and culled using Gblocks (23) with the following settings: -b1=(n/2)+1 -b2= (n/2)+1 -b3= (n/2) -b4=2 -b5=h, where n=number of sequences in the alignment (42 for Roseobacter and 22 for SAR11). These alignments were then concatenated using the normalizeAlignments.py and catPhylip.pl scripts available as associated files. Phylogenetic inference was performed with RAxML HPC v7.7.6 using the gamma model for rate heterogeneity with optimized substitution rates, the LG amino acid substitution matrix (24), and 1000 bootstrap resamplings. Phylogenetic trees were rooted at HTCC2255 for *Roseobacter* and HIMB59 for SAR11.

Phylogenomic structures of biological traits: Phylogenetic signal' or 'phylogenetic structuring' are used in this study to refer to traits (in this case the ability to acquire various metals) whose presence among taxa in a phylogeny are autocorrelated with the structure of the phylogeny. We used the τ_D statistic (trait depth) from the consenTRAIT algorithm and Fritz and Purvis' D for phylogenetic dispersion to

predict the extent with which Roseobacter and SAR11 phylogeny explains the distribution of metal uptake categories in each genome. D was calculated using the CAPER package in R with 1000 random permutations under a model of Brownian motion for discrete trait evolution, where D < 0 indicates strong trait clustering, D = 0 indicates Brownian evolution, D = 1 indicates random evolution, and D > 1 indicates over-dispersion. D was calculated in the R package 'caper' using the highest scoring maximum likelihood tree from Roseobacter and SAR11. R Command: phylo.d(caper_object, binvar=TRACEMETAL_TRAIT). D values were considered significant if the probability of matching a random distribution ($P(D)_{random}$) was smaller than 5% ($P(D)_{random} < 0.05$).

The τ_D statistic was estimated as the average amino acid distance in substitutions between leaves and root node of a clade carrying a particular trait, whereas clades are defined by 90% of leaves possessing a metal uptake trait. Traits without any neighbors were scored using half the distance to the nearest internal node. We considered a particular τ_D value as significant ($\alpha = 0.05$) when fewer than 5% of τ_D values resulting from 1000 random permutation of taxa across the tree (10 permutations per bootstrap) were greater than or equal to the true τ_D value. We did not calculate D and τ_D for metal uptake traits present in all genomes because they are completely phylogenetically conserved. Trait depth (τ_D) was calculated using a custom R script provided with the original description of the consenTRAIT algorithm(8). The script was modified to include a random permutation procedure for significance testing. Briefly, for each phylogenetic tree in the multi-tree boostrap file the distributions of binary traits was randomly shuffled ten times for each tree resulting in 10X the individual trait depth calculation performed in the original script. The proportion of those calculations greater than the value calculated for the best maximum likelihood tree is then equal to the *P* value (proportion of values greater than that observed by chance alone). Custom scripts for calculating this measure are available in the supplemental material scripts.tar.gz file.

Ordination: To quantify the spread of SAR11 and *Roseobacter* genomes in the ordination, the permdisp2 procedure from VEGAN was used to look at multivariate homogeneity of group dispersions for each taxonomic class. Significance of group dispersion magnitude was assessed using permutation analysis, and the test was performed to account for unequal sample size. Effect sizes of differences in dispersion between *Roseobacter* and SAR11 were assessed using Cohen's D, while significance was assessed using a Mann-Whitney *U* test. We fit environmental categorical factors corresponding to ocean basin of isolation, surface attached versus planktonic lifestyle, and coastal versus pelagic isolation location to the PCA ordination and tested whether correlation was significant. We also tested whether a variety of numerical *Roseobacter* genome features were significantly associated with the full ordination.

Statistical analyses: To explore specific statistical association between genome features and metal transporter inventory, we performed pairwise Spearman's rank correlation tests between each metal transporter and the numerical genome features for the combined *Roseobacter* and SAR11 dataset (Fig. S3A) and *Roseobacter* alone (Fig. S3B). Correlation networks were visualized using Cytoscape. R scripts performing all statistical calculations are available as markdown documents in the supplemental material scripts.tar.gz file.

Table S1: Metal transport components and their frequency of occurrence

Name	Function	Metal(s)
hupE/ureJ	Predicted secondary inner membrane Ni transporter, associated with hydrogenase/urease	Ni^{2+}
nikA	Type II solute binding protein	Ni^{2+}
corA	Inner membrane ion channel	Co^{2+}, Ni^{2+}, Mg
cbiM	Inner membrane permease	Co^{2+}
cbiQ	Inner membrane permease	Co^{2+}
cbtA	Predicted inner membrane channel	Co^{2+}
cbtB	Predicted inner membrane channel	Co^{2+}
btuF	TroA family solute binding protein	Co^{2+} as Vitamin B_{12}
copZ	Heavy metal binding domain with N-terminal Cu-interacting ATPase	$Cu^{+/2+}$ and other heavy metals
copA	P-type IB ATPase - Cu import and efflux	$Cu^{+/2+}$ and other heavy metals
NRAMP	Inner membrane permease	Fe^{2+}, Mn^{2+}
ZIP	Inner membrane permease	Fe^{2+}, Zn^{2+}
psaA	troA family solute binding protein	Mn^{2+}
hemV2	troA family solute binding protein	Mn^{2+}, Zn^{2+}
troA-a	troA family solute binding protein	Mn^{2+}, Zn^{2+}
troA-f	troA family solute binding protein	Mn^{2+}, Zn^{2+}
znuA	troA family solute binding protein	Zn^{2+}
afuA	Type II periplasmic binding family	Fe^{3+}
PBP2 Fbp-like 1	Type II periplasmic binding family	Fe^{3+}
PBP2 Fbp-like 2	Type II periplasmic binding family	Fe^{3+}
PBP2 Fbp-like 3	Type II periplasmic binding family	Fe^{3+}
PBP2 futA-like	Type II periplasmic binding family	Fe^{3+}
feoB	Inner membrane permease	Fe^{2+}
FTR1	Inner membrane permease	Fe^{2+}
hemS	Cytoplasmic Oxygenase/Chaperone	Heme/Hemoglobin
hutB	troA family solute binding protein	Heme/Hemoglobin
TBDT MCL1	TonB-dependent transporter	Heme/Hemoglobin
fatB	troA family solute binding protein	Catecholate Siderophores
TBDT MCL2	TonB-dependent transporter	Catecholate Siderophores
fhuD	troA family solute binding protein	Hydroxamate Siderophores
TBDT MCL3	TonB-dependent transporter	Hydroxamate Siderophores
fepB	troA family solute binding protein	Mixed / multiple siderophores
TBDT MCL4	TonB-dependent transporter	Mixed / multiple siderophores
entE	Siderophore biosynthesis	Siderophore biosynthesis (NIS or NRPS)
entF	Siderophore biosynthesis	Siderophore biosynthesis (NIS or NRPS)
rbhC	Siderophore biosynthesis	Siderophore biosynthesis (NIS or NRPS)
SIP	Cytoplasmic ferric reductase	Siderophore processing
TBDT MCL5	TonB-dependent transporter	Unknown
TBDT MCL6	TonB-dependent transporter	Unknown
TBDT MCLnull	TonB-dependent transporter	Unknown / siderophores
troA superfamily	Solute binding protein assignable only to troA superfamily	Unknown / Multiple

"Function" designates the molecular role of each gene/family, while "Metal(s)" indicates the metal or metal complex with which each family is known or predicted to interact.

Table S2: TBDT Markov Clusters and their occurrence in Roseobacter genomes

Cluster	Substrate Transported	Cluster Abun.	Freq.	Fur-box Freq.	Gene enrichment Gene/PFAM	*P* value	Odds Ratio	Cluster Frac.	Avg. Gene Dist.
MCL1	Heme	19	45%	85%	*hmuS*	4.0×10^{-45}	4225	0.95	1.29
					hutB	1.3×10^{-35}	5973	0.95	2.14
					FecCD	7.6×10^{-32}	94	1.1	4
					ExbD	1.8×10^{-22}	68	0.84	4.2
					TonB_2	3.9×10^{-19}	240	0.53	4.7
					MotA_ExbB	2.5×10^{-17}	54	0.67	3.2
					DUF4178	5.3×10^{-8}	189	0.21	8.5
MCL2	Catecholate Siderophores	18	26%	33%	*fatB*	3.6×10^{-19}	1332	0.56	2.25
					FecCD	2.9×10^{-34}	105	1.2	3.77
					DUF2218	2.7×10^{-15}	511	0.39	3.57
					MarR_2	4.8×10^{-11}	58	0.61	2
MCL3	Hydroxamate Siderophores	18	24%	55%	*fhuD*	1.5×10^{-8}	287	0.44	1
					FecCD	7.1×10^{-16}	51	0.67	2.3
					SIP	1.3×10^{-12}	148	0.33	3
					FAD_binding_9	1.8×10^{-10}	116	0.33	3
					HTH_18	1.5×10^{-8}	10	0.67	1
					ABC_membrane	2.7×10^{-6}	11	0.44	3.83
MCL4	Mixed Siderophores	6	14%	33%	FecCD	5.5×10^{-26}	199	2.33	6.36
					ExbD	1.5×10^{-21}	165	2	3.5
					SIP	2.9×10^{-13}	376	1	5
					FAD_binding_9	3.4×10^{-13}	364	1	5
					Peripla_BP_2	5.5×10^{-12}	110	1.16	7.42
					TonB_2	5.8×10^{-11}	313	0.83	1
					MotA_ExbB	1.4×10^{-9}	75	1	4.5
MCL5	Unknown – Iron?	5	12%	60%	TonB_C	2.5×10^{-10}	1784	0.8	4
					2OG-FeII_Oxy_3	2.3×10^{-8}	206	0.8	1
					FMN_dh	1.9×10^{-6}	63	0.8	5
					MotA_ExbB	2.5×10^{-6}	59	0.8	2
					ExbD	2.5×10^{-6}	58	0.8	3
					hemP	4.6×10^{-6}	138	0.6	3.3
MCL6	Unknown	4	10%	0%	Peripla_BP_2	5.0×10^{-7}	90	1	1
					TatD_DNase	2.6×10^{-6}	170	0.75	4.7
MCLnull	Mostly unknown, siderophore?	11	14%	27%	NA	NA	NA	NA	NA

TBDT sequence similarity clusters, their putatively ascribed function, and gene/PFAM enrichment for each cluster. “Cluster Abun.” is the total number of TBDTs in each cluster. “Freq.” indicates the percentage of Roseobacter genomes that contain at least one of the TBDTs from a given cluster. “Fur-box Freq.” denotes the percentage of TBDT in the cluster with a detectable upstream Fur-box binding motif. “Gene/PFAM” are genes from Table 1 and/or additional PFAMs that are enriched in the neighborhood of each TBDT cluster. Specific gene groups from Table 1 are listed preferentially over their parent PFAM superfamilies when both are significantly enriched (e.g., *hutB* is listed for MCL1 instead of its parent protein family Peripla_BP_2). For enriched genes/PFAMs, “p value” indicates the significance of enrichment (see Materials and Methods), “Odds Ratio” is the frequency of the gene/PFAM in the TBDT neighborhood divided by the frequency outside the

neighborhood, “Cluster Frac.” is the total abundance of a gene/PFAM inside all neighborhoods of a particular cluster divided by the total number of TBDTs in that cluster, and “Avg. Gene Dist.” is the average number of genes separating a TBDT of the given cluster and the respective gene/PFAM

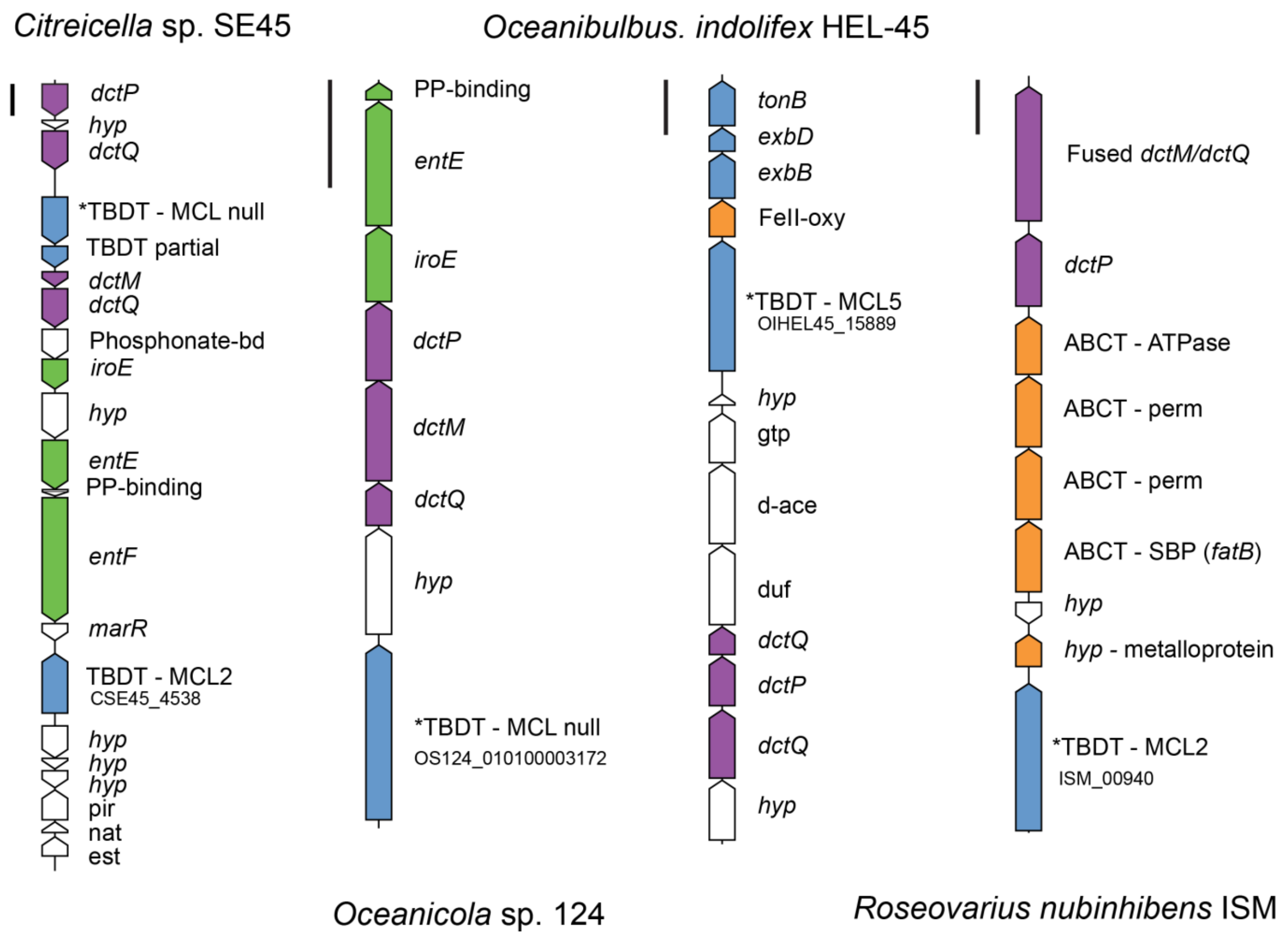

Supplemental Fig. S1: Examples of TRAP transporters in genome neighborhoods with genes involved in siderophore biosynthesis or siderophore-like compound uptake. Purple arrows are TRAP genes, green arrows represent genes likely involved in siderophore biosynthesis, light blue arrows represent TBDTs and associated energy transduction components, and orange arrows represent other genes related to Fe uptake and metabolism. Genes labeled with an asterisk have an upstream Fur box motif. The locus IDs of four TBDT are provided for reference. Scale bars in the upper left corner of each gene group represent 1000 base pairs. PP-binding, phosphopantetheine attachment site; entE, enterobactin synthase subunit E; entF, enterobactin synthase subunit F; iroE, putative enterobactin esterase; Phosphonate-bd, ABCT phosphonate SBP; dctQ, TRAP large permease component; dctM, TRAP small permease component; dctP, TRAP periplasmic component; marR, transcriptional regulator; pir, pirin-like Fe2+ containing protein; nat, GCN5-related N-acetyl-transferase; est, predicted esterase; tonB, periplasmic protein TonB; exbB, biopolymer transport protein exbD/tolR; exbD, motA/tolQ/exbB proton channel family; FeII-oxy, Fe2+ dependent oxygenase; gtp, putative GTPases (G3E family); d-ace, D-aminoacylases (N-acyl-D-Amino acid amidohydrolases); duf, protein of unknown function (DUF1485); ABCT, ATP Binding Cassette Transporter; perm, IM permease; SBP, inner membrane solute binding protein.

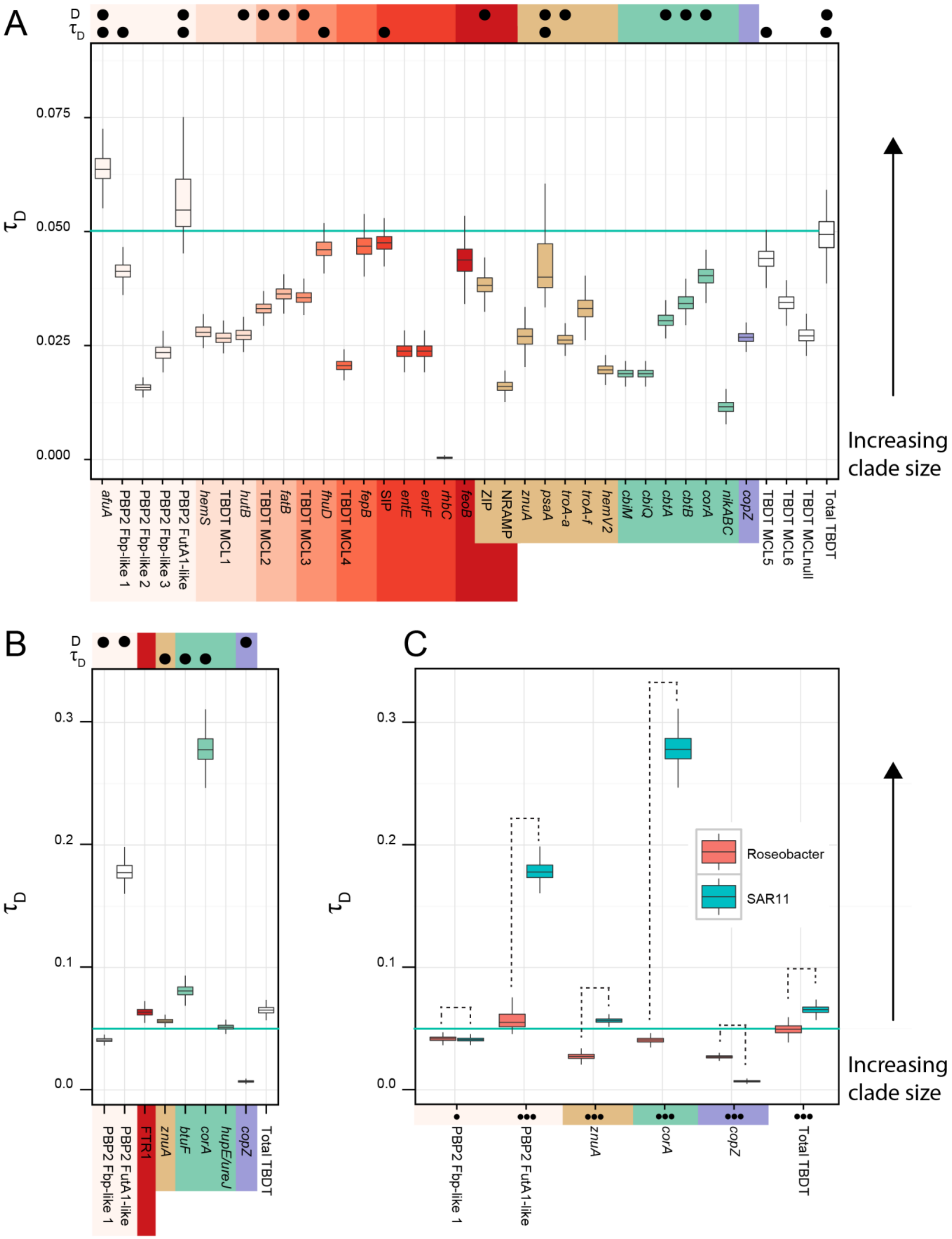

A
B
C
D
τ_D
0.075
0.050
0.025
0.000
0.3
0.2
0.1
0.0
Increasing clade size
afuA
PBP2 Fbp-like 1
PBP2 Fbp-like 2
PBP2 Fbp-like 3
PBP2 FutA1-like
hemS
TBDT MCL1
hutB
TBDT MCL2
fatB
TBDT MCL3
fhuD
TBDT MCL4
fepB
SIP
entE
entF
rhbC
feoB
ZIP
NRAMP
znuA
psaA
troA-a
troA-f
hemV2
cbiM
cbiQ
cbtA
cbtB
corA
nikABC
copZ
TBDT MCL5
TBDT MCL6
TBDT MCLnull
Total TBDT
FTR1
btuF
hupE/ureJ
Roseobacter
SAR11

Supplemental Fig. S2: Boxplot showing trait depth (τ_D) of metal uptake genes with respect to **(A)** Roseobacter and **(B)** SAR11 phylogenies. Black circles above plots in **(A)** and **(B)** denote non-random phylogenetic distribution as assessed by the consenTRAIT algorithm ($P < 0.1$) and the independent D metric for phylogenetic dispersion of Fritz and Purvis ($P(D)_{random} < 0.05$). **(C)** displays differences in trait depth for metal uptake traits shared between the SAR11 and roseobacter groups. Each comparison is denoted with dashed lines and all differences in mean are significant (Student's *t*-test, $P < 0.05$). Dots above transporter labels indicate effect size calculated using Cohen's D. Single dot represents a small effect size (D < 0.5), while three dots represents a large effect size (D > 1). Transporters are colored as in Fig. 1. For visual reference, a teal line is marked at τ_D of 0.05 in each panel that corresponds to the maximum value for subtrees marked in Fig. 1. The box denotes positions of upper and lower quartiles, the middle crossbar represents median value of the group, lines are extended to values that are within 1.5X of the inter-quartile range. Outliers are not displayed for clarity but included in statistical tests.

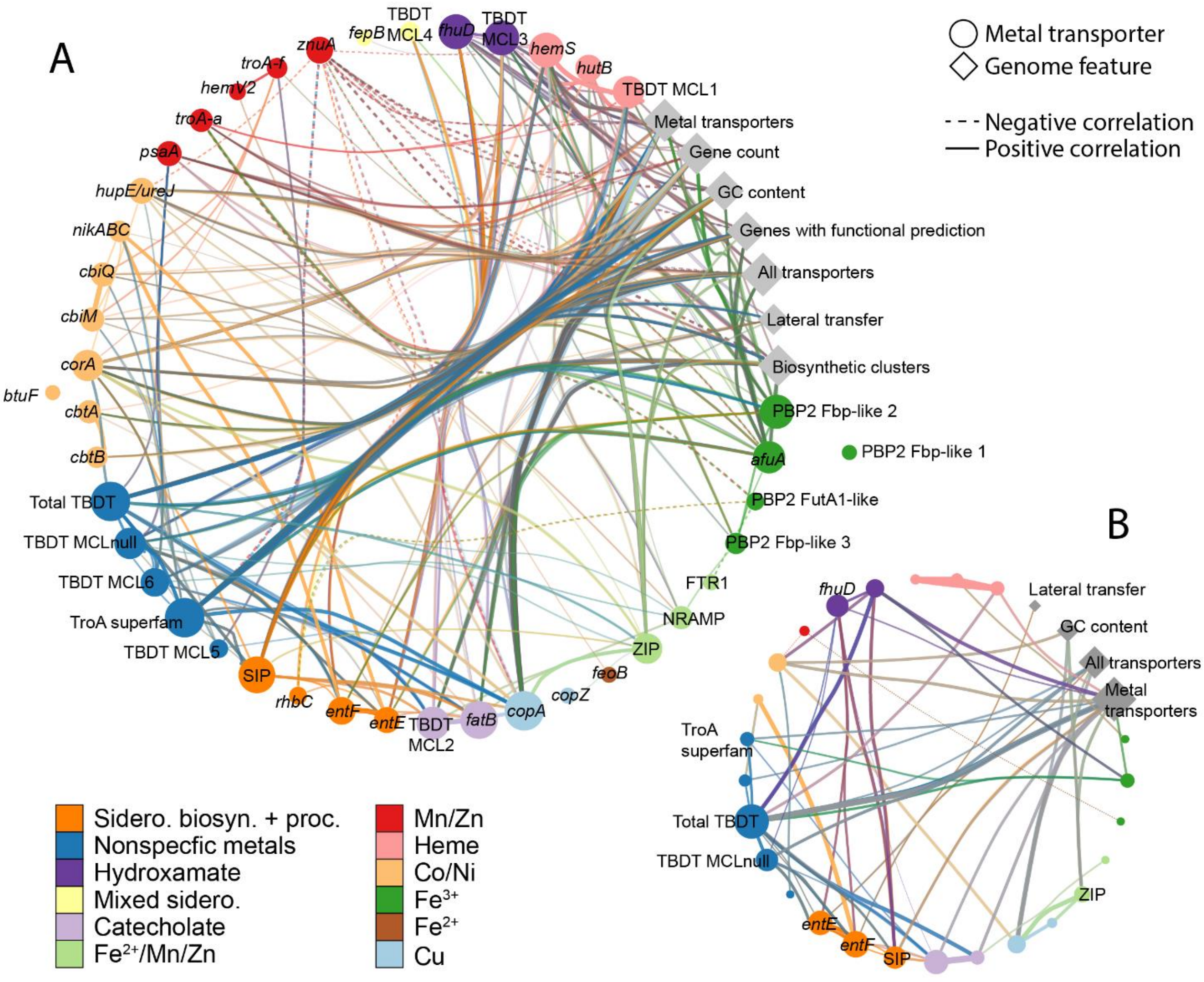

Supplemental Fig. S3: Network visualization of pairwise Spearman's rank correlation coefficients (ρ) between metal uptake genes (circular nodes) and genome features (gray diamond nodes). (A) represents ρ values calculated from a combined SAR11 and Roseobacter dataset (N = 64), while (B) is calculated from Roseobacter only (N=42). Nodes are colored based on the category of metal uptake, and their size is proportional to the number of linked edges. Edges represent correlation coefficients between variables with $P < 0.05$. Solid edges represent positive correlations while dashed edges represent negative correlation. Edges are colored corresponding to nodes. Edge thickness and opacity is proportional to the magnitude of the correlation coefficient. Network layout was calculated using the attribute layout in Cytoscape and edges are bundled for clarity. In (B) singleton nodes and labels for some nodes are omitted for clarity. Numerical ρ and P values are available in the supplementary material.

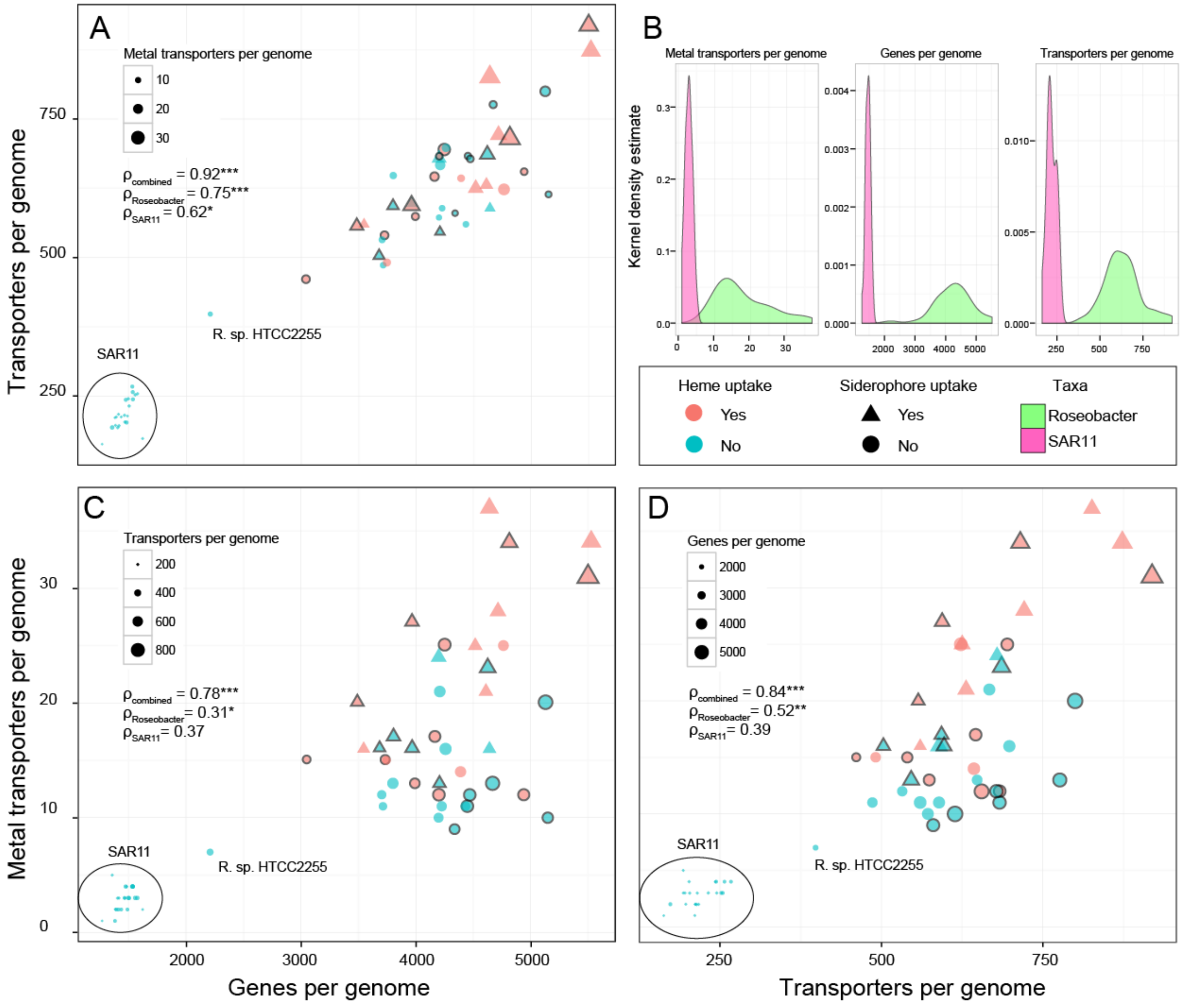

Supplemental Fig. S4: Scatterplots displaying pairwise comparisons of three different genome features (genes per genome, transporters per genome, and metal transporters per genome) determined from the 62 SAR11 and Roseobacter genomes. In **(A)**, **(C)**, and **(D)** each point represents one genome and is colored according to the genomic potential for heme uptake, shaped according to the genomic potential for siderophore uptake, and sized based on the genome feature omitted from the bivariate comparison. Points with a black outline have been experimentally confirmed to be particle associated or were isolated from an abiotic or biotic surface. The SAR11 genomes circled in black and the HTCC225 genome are the only genomes with confirmed planktonic lifestyles. In each scatterplot, Spearman's rank correlation coefficients (ρ) are displayed for SAR11 genomes only (N= 22), Roseobacter genomes only (N = 42), and the combined genomes from each group (N=64). Asterisks denote statistical significance; *** P<1e^{-10}, ** P<1e^{-3}, * P<0.05. **(B)** displays kernel density estimates for each of **(A,C and D)** and is colored by SAR11 and Roseobacter groups.

Supplemental References

1. **Dupont CL**, **Rusch DB**, **Yooseph S**, **Lombardo M-J**, **Alexander Richter R**, **Valas R**, **Novotny M**, **Yee-Greenbaum J**, **Selengut JD**, **Haft DH**, **Halpern AL**, **Lasken RS**, **Nealson K**, **Friedman R**, **Craig Venter J**. 2012. Genomic insights to SAR86, an abundant and uncultivated marine bacterial lineage. ISME J **6**:1186–1199.

2. **Fernández-Gómez B**, **Richter M**, **Schüler M**, **Pinhassi J**, **Acinas SG**, **González JM**, **Pedrós-Alió C**. 2013. Ecology of marine Bacteroidetes: a comparative genomics approach. ISME J **7**:1026–1037.

3. **Mulligan C**, **Fischer M**, **Thomas GH**. 2011. Tripartite ATP-independent periplasmic (TRAP) transporters in bacteria and archaea. FEMS Microbiol Rev **35**:68–86.

4. **Schauer K**, **Rodionov D**, **de Reuse H**. 2008. New substrates for TonB-dependent transport: do we only see the "tip of the iceberg"? Trends Biochem Sci **33**:330–338.

5. **Mulligan C**, **Geertsma ER**, **Severi E**, **Kelly DJ**, **Poolman B**, **Thomas GH**. 2009. The substrate-binding protein imposes directionality on an electrochemical sodium gradient-driven TRAP transporter. Proc Natl Acad Sci U S A **106**:1778–1783.

6. **Yoshizawa S**, **Kumagai Y**, **Kim H**, **Ogura Y**, **Hayashi T**, **Iwasaki W**, **DeLong EF**, **Kogure K**. 2014. Functional characterization of flavobacteria rhodopsins reveals a unique class of light-driven chloride pump in bacteria. Proc Natl Acad Sci U S A **111**:6732–6737.

7. **Sandy M**, **Butler A**. 2009. Microbial iron acquisition: marine and terrestrial siderophores. Chem Rev **109**:4580–4595.

8. **Martiny AC**, **Treseder K**, **Pusch G**. 2013. Phylogenetic conservatism of functional traits in microorganisms. ISME J **7**:830–838.

9. **Fritz S a.**, **Purvis A**. 2010. Selectivity in mammalian extinction risk and threat types: A new measure of phylogenetic signal strength in binary traits. Conserv Biol **24**:1042–1051.

10. **Hopkinson B**, **Barbeau K**. 2012. Iron transporters in marine prokaryotic genomes and metagenomes. Environ Microbiol **14**:114–128.

11. **Saier M**, **Reddy V**, **Tamang D**, **Västermark Å**. 2014. The transporter classification database. Nucleic Acids Res **42**:251–258.

12. **Cordero OX**, **Ventouras L-A**, **DeLong EF**, **Polz MF**. 2012. Public good dynamics drive evolution of iron acquisition strategies in natural bacterioplankton populations. Proc Natl Acad Sci U S A **109**:20059–20064.

13. **Luo H**, **Löytynoja A**, **Moran MA**. 2012. Genome content of uncultivated marine Roseobacters in the surface ocean. Environ Microbiol **14**:41–51.

14. **Biers EJ**, **Sun S**, **Howard EC**. 2009. Prokaryotic genomes and diversity in surface ocean waters: interrogating the global ocean sampling metagenome. Appl Environ Microbiol **75**:2221–2229.

15. **Altschul SF**, **Madden TL**, **Schäffer A a.**, **Zhang J**, **Zhang Z**, **Miller W**, **Lipman DJ**. 1997. Gapped BLAST and PSI-BLAST: a new generation of protein database search programs. Nucleic Acids Res **25**:3389–3402.

16. **Eddy SR**. 2011. Accelerated Profile HMM Searches. PLoS Comput Biol **7**:e1002195.

17. **Crosa JH**, **Walsh CT**. 2002. Genetics and assembly line enzymology of siderophore biosynthesis in

bacteria. Microbiol Mol Biol Rev **66**:223–249.

18. **Winterberg B**, **Uhlmann S**, **Linne U**, **Lessing F**, **Marahiel M a.**, **Eichhorn H**, **Kahmann R**, **Schirawski J**. 2010. Elucidation of the complete ferrichrome A biosynthetic pathway in Ustilago maydis. Mol Microbiol **75**:1260–1271.

19. **Morris JH**, **Apeltsin L**, **Newman AM**, **Baumbach J**, **Wittkop T**, **Su G**, **Bader GD**, **Ferrin TE**. 2011. clusterMaker: a multi-algorithm clustering plugin for Cytoscape. BMC Bioinformatics **12**:436.

20. **Rodionov D**, **Gelfand M**, **Todd J**, **Curson A**, **Johnston A**. 2006. Computational reconstruction of iron- and manganese-responsive transcriptional networks in alpha-proteobacteria. PLoS Comput Biol **2**:e163.

21. **Baichoo N**, **Helmann JD**. 2002. Recognition of DNA by Fur: a reinterpretation of the Fur box consensus sequence. J Bacteriol **184**:5826–5832.

22. **Edgar RC**. 2004. MUSCLE: Multiple sequence alignment with high accuracy and high throughput. Nucleic Acids Res **32**:1792–1797.

23. **Castresana J**. 2000. Selection of conserved blocks from multiple alignments for their use in phylogenetic analysis. Mol Biol Evol **17**:540–552.

24. **Le SQ**, **Gascuel O**. 2008. An improved general amino acid replacement matrix. Mol Biol Evol **25**:1307–1320.
